# Supplementary figures and images for: Linkage disequilibrium and population structure in a core collection of Brassica napus (L.)
Source: PLoS One. 2022 Mar 1;17(3):e0250310. doi: 10.1371/journal.pone.0250310 (PMC8887726; doi:10.1371/journal.pone.0250310)

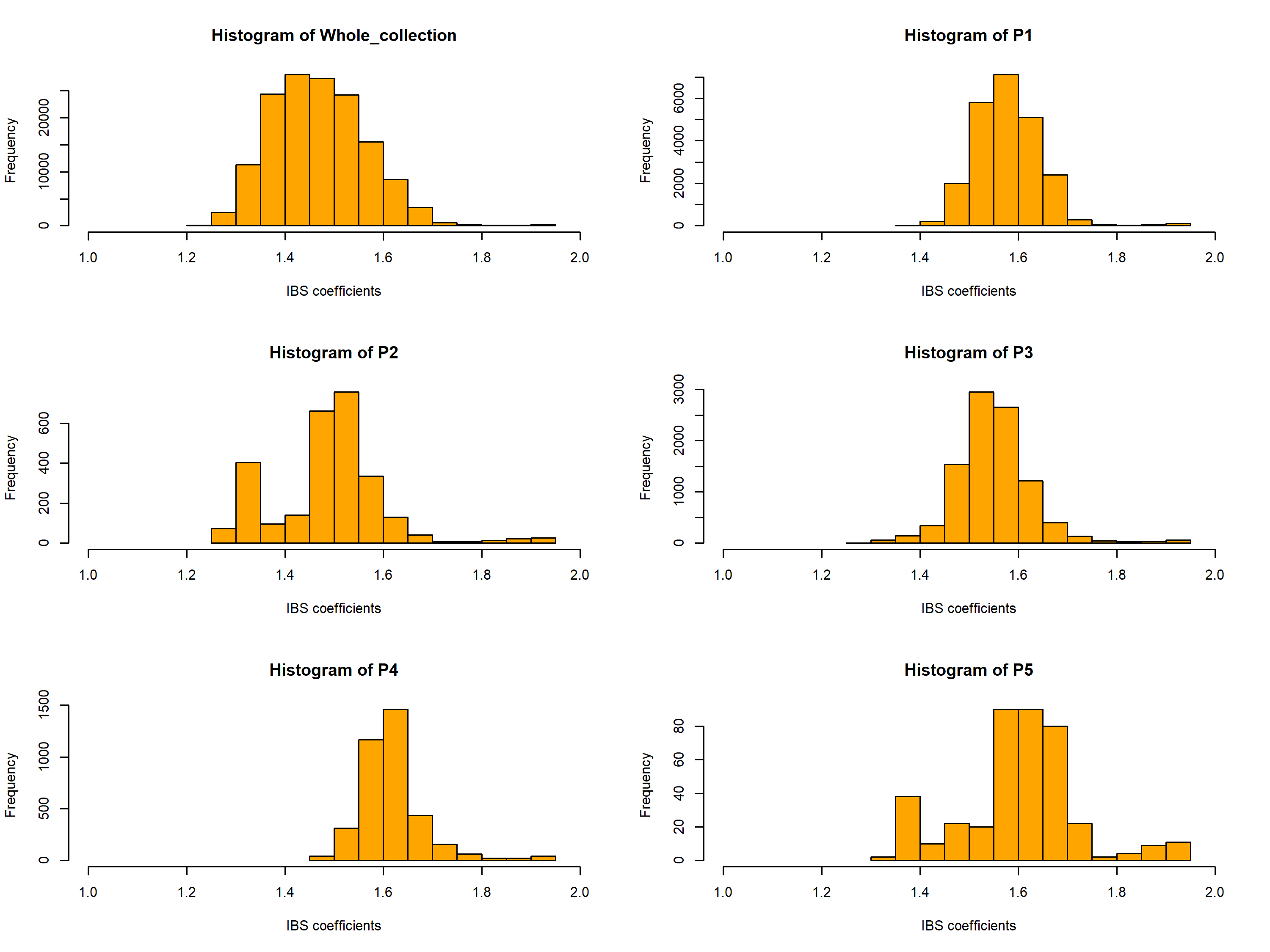

Supplement: S1 Fig — (TIFF) [file pone.0250310.s008.tiff]

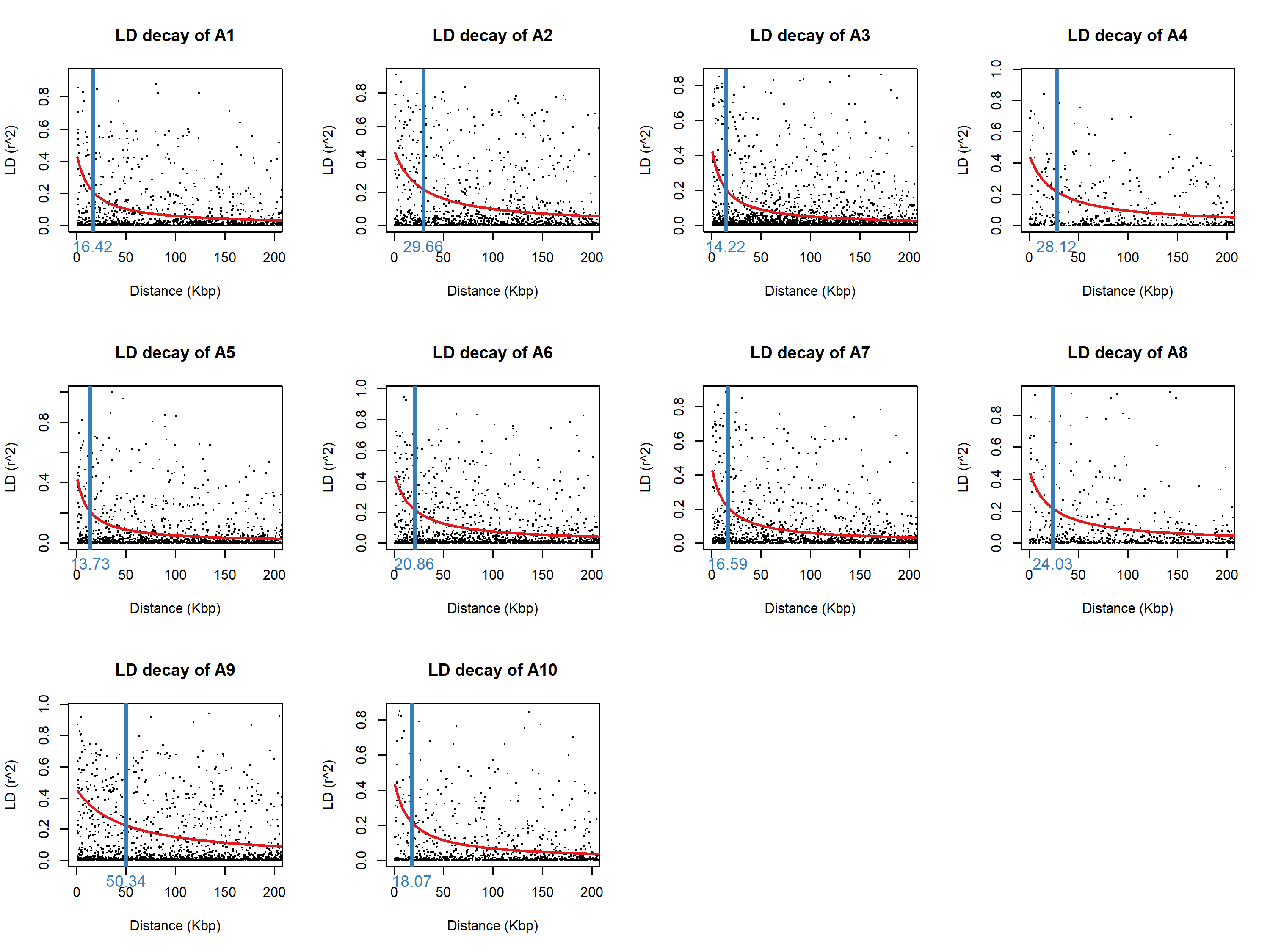

Supplement: S2 Fig — (TIFF) [file pone.0250310.s009.tiff]

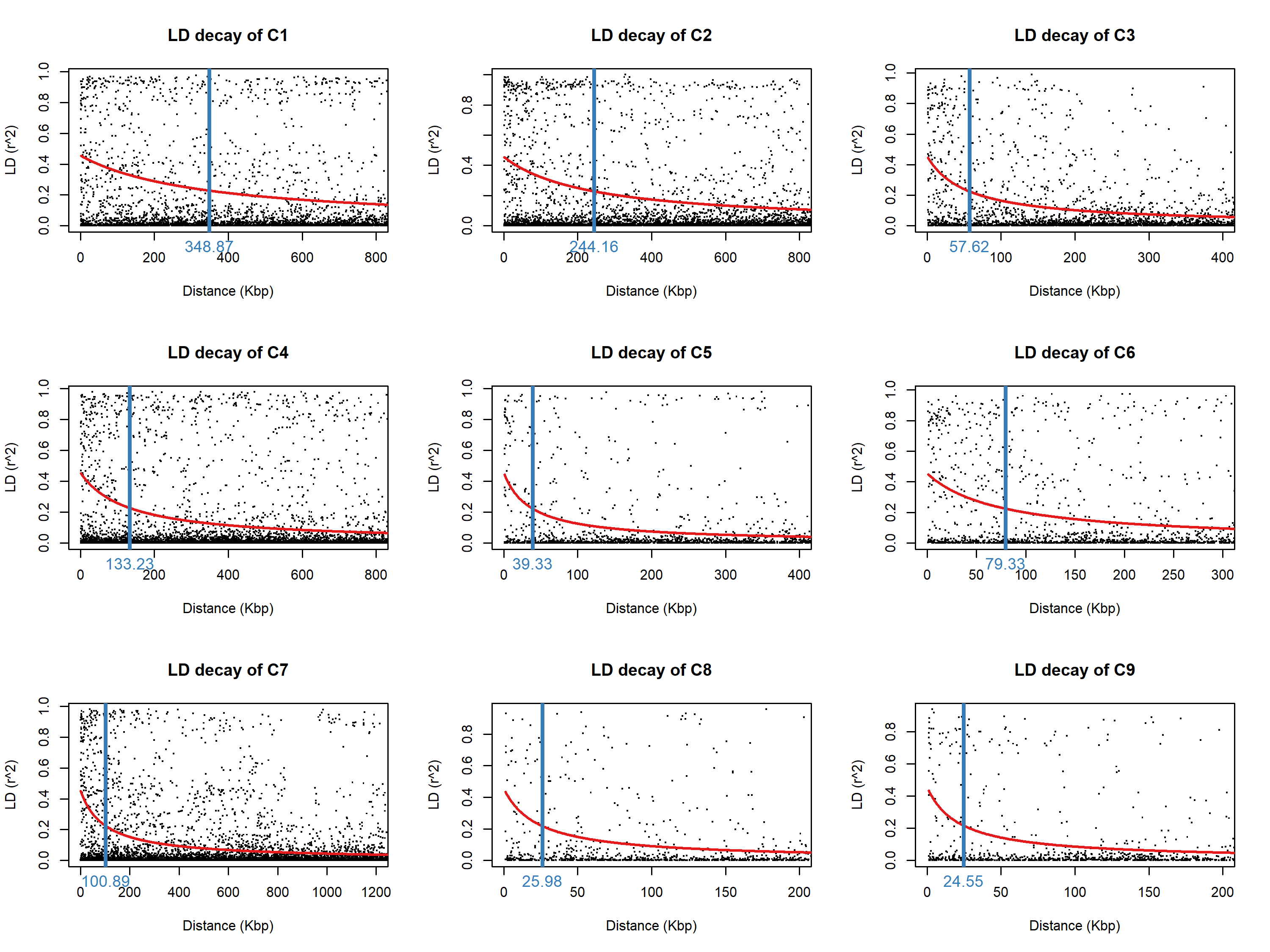

Supplement: S3 Fig — (TIFF) [file pone.0250310.s010.tiff]
