# Supplementary material for: Linkage disequilibrium and population structure in a core collection of Brassica napus (L.)
Source: PLoS One. 2022 Mar 1;17(3):e0250310. doi: 10.1371/journal.pone.0250310 (PMC8887726; doi:10.1371/journal.pone.0250310)
Supplement: Attachment — Submitted filename: PONE-D-21-09997_reviewer.pdf [file pone.0250310.s011.pdf]

PLOS ONE

Linkage disequilibrium and population structure in a core collection of Brassica napus (L.)

--Manuscript Draft--

**Manuscript Number:**	PONE-D-21-09997
**Article Type:**	Research Article
**Full Title:**	Linkage disequilibrium and population structure in a core collection of Brassica napus (L.)
**Short Title:**	LD and population structure Brassica napus
**Corresponding Author:**	Mukhlesur Rahman, Ph.D.<br>North Dakota State University<br>Fargo, UNITED STATES
**Keywords:**	LD, population structure, Brassica napus
**Abstract:**	Estimation of genetic diversity in rapeseed/canola is important for sustainable breeding program to provide an option for the development of new breeding lines. The objective of this study was to elucidate the patterns of genetic diversity within and among different structural groups, and measure the extent of linkage disequilibrium (LD) of 383 globally distributed rapeseed/canola germplasm using 8,502 single nucleotide polymorphism (SNP) markers. The germplasm accessions were divided into five subpopulations (P1 to P5) with obvious geographic and growth habit-related patterns. All subpopulations showed moderate genetic diversity (average H = 0.22 and I = 0.34). The pairwise F st comparison revealed a great degree of divergence (F st > 0.24) between most of the combinations. The rutabaga type showed highest divergence with spring and winter types. Higher divergence was also found between winter and spring types. Overall, mean linkage disequilibrium was 0.03 and it decayed to its half maximum within < 45 kb distance for whole genome. The LD decay was slower in C genome (< 93 kb), relative to the A genome (< 21 kb) which was confirmed by availability of larger haplotype blocks in C genome than A genome. To maximize genetic gain, hybridization between rutabaga and other types are potentially the best option. Hybridization between spring and winter, semi-winter type is also helpful to maximize the diversity in subsequent populations. Low genetic differentiation between both spring type subpopulations (P4 and P3) will accelerate favorable allele accumulation for specific traits in elite lines. The Neighbor-Joining tree and kinship matrix will assist to identify distantly related genotypes from subpopulations to utilize in hybridization. The low levels of LD and population structure make the core collection an important resource for association mapping efforts to identify genes useful in crop improvement as well as for selection of parents for hybrid breeding.
**Order of Authors:**	Mukhlesur Rahman, Ph.D.
	Ahasanul Hoque
	Jayanta Roy
**Additional Information:**	
**Question**	**Response**
**Financial Disclosure**<br><br>Enter a financial disclosure statement that describes the sources of funding for the work included in this submission. Review the submission guidelines for detailed requirements. View published research articles from *PLOS ONE* for specific examples.	The funders had no role in study design, data collection and analysis, decision to publish, or preparation of the manuscript.

Powered by Editorial Manager® and ProduXion Manager® from Aries Systems Corporation

This statement is required for submission and **will appear in the published article** if the submission is accepted. Please make sure it is accurate.<br><br>**Unfunded studies**<br>Enter: *The author(s) received no specific funding for this work.*<br><br>**Funded studies**<br>Enter a statement with the following details:<br>• Initials of the authors who received each award<br>• Grant numbers awarded to each author<br>• The full name of each funder<br>• URL of each funder website<br>• Did the sponsors or funders play any role in the study design, data collection and analysis, decision to publish, or preparation of the manuscript?<br>• **NO** - Include this sentence at the end of your statement: *The funders had no role in study design, data collection and analysis, decision to publish, or preparation of the manuscript.*<br>• **YES** - Specify the role(s) played.<br><br>* typeset	
**Competing Interests**<br><br>Use the instructions below to enter a competing interest statement for this submission. On behalf of all authors, disclose any competing interests that could be perceived to bias this work—acknowledging all financial support and any other relevant financial or non-financial competing interests.<br><br>This statement is required for submission and **will appear in the published article** if the submission is accepted. Please make sure it is accurate and that any funding sources listed in your Funding Information later in the submission form are also declared in your Financial Disclosure statement.<br><br>View published research articles from *PLOS ONE* for specific examples.	The authors have declared that no competing interests exist.

**NO authors have competing interests**<br><br>Enter: *The authors have declared that no competing interests exist.*<br><br>**Authors with competing interests**<br><br>Enter competing interest details beginning with this statement:<br><br>*I have read the journal's policy and the authors of this manuscript have the following competing interests: [insert competing interests here]*<br><br>* typeset	
**Ethics Statement**<br><br>Enter an ethics statement for this submission. This statement is required if the study involved:<br><br>• Human participants<br>• Human specimens or tissue<br>• Vertebrate animals or cephalopods<br>• Vertebrate embryos or tissues<br>• Field research<br><br>Write "N/A" if the submission does not require an ethics statement.<br><br>General guidance is provided below. Consult the submission guidelines for detailed instructions. **Make sure that all information entered here is included in the Methods section of the manuscript.**	N/A

Powered by Editorial Manager® and ProduXion Manager® from Aries Systems Corporation

**Format for specific study types**<br><br>**Human Subject Research (involving human participants and/or tissue)**<br>• Give the name of the institutional review board or ethics committee that approved the study<br>• Include the approval number and/or a statement indicating approval of this research<br>• Indicate the form of consent obtained (written/oral) or the reason that consent was not obtained (e.g. the data were analyzed anonymously)<br><br>**Animal Research (involving vertebrate animals, embryos or tissues)**<br>• Provide the name of the Institutional Animal Care and Use Committee (IACUC) or other relevant ethics board that reviewed the study protocol, and indicate whether they approved this research or granted a formal waiver of ethical approval<br>• Include an approval number if one was obtained<br>• If the study involved *non-human primates*, add *additional details* about animal welfare and steps taken to ameliorate suffering<br>• If anesthesia, euthanasia, or any kind of animal sacrifice is part of the study, include briefly which substances and/or methods were applied<br><br>**Field Research**<br><br>Include the following details if this study involves the collection of plant, animal, or other materials from a natural setting:<br>• Field permit number<br>• Name of the institution or relevant body that granted permission	
**Data Availability**<br><br>Authors are required to make all data underlying the findings described fully available, without restriction, and from the time of publication. PLOS allows rare exceptions to address legal and ethical concerns. See the PLOS Data Policy and FAQ for detailed information.	Yes - all data are fully available without restriction

A Data Availability Statement describing where the data can be found is required at submission. Your answers to this question constitute the Data Availability Statement and **will be published in the article**, if accepted.<br><br>**Important:** Stating 'data available on request from the author' is not sufficient. If your data are only available upon request, select 'No' for the first question and explain your exceptional situation in the text box.<br><br>Do the authors confirm that all data underlying the findings described in their manuscript are fully available without restriction?	
**Describe where the data may be found in full sentences. If you are copying our sample text, replace any instances of XXX with the appropriate details.**<br><br>• If the data are **held or will be held in a public repository**, include URLs, accession numbers or DOIs. If this information will only be available after acceptance, indicate this by ticking the box below. For example: *All XXX files are available from the XXX database (accession number(s) XXX, XXX.*).<br>• If the data are all contained **within the manuscript and/or Supporting Information files**, enter the following: *All relevant data are within the manuscript and its Supporting Information files.*<br>• If neither of these applies but you are able to provide **details of access elsewhere**, with or without limitations, please do so. For example:<br><br>*Data cannot be shared publicly because of [XXX]. Data are available from the XXX Institutional Data Access / Ethics Committee (contact via XXX) for researchers who meet the criteria for access to confidential data.*<br><br>*The data underlying the results presented in the study are available from (include the name of the third party*	All row sequence data and variant data are available in NCBI and ENA repository. The accession id for them are PRJNA687906 (https://www.ncbi.nlm.nih.gov/biosample/17159566) and PRJEB42419 (https://www.ebi.ac.uk/eva/?eva-study=PRJEB42419), respectively. The other datasets used and/or analyzed during the current study are available from the corresponding author on reasonable request.

and contact information or URL).<br>• This text is appropriate if the data are owned by a third party and authors do not have permission to share the data.<br><br>* typeset	
Additional data availability information:	Tick here if the URLs/accession numbers/DOIs will be available only after acceptance of the manuscript for publication so that we can ensure their inclusion before publication.

Manuscript Click here to access/download;Manuscript;Manuscript.docx

Linkage disequilibrium and population structure in a core collection of *Brassica napus* (L.)

Mukhlesur Rahman[1¶*], Ahasanul Hoque[1,2¶], Jayanta Roy[1]

[1]Department of Pant Sciences, North Dakota State University, Fargo, North Dakota, USA

[2]Department of Genetics and Plant Breeding, Bangladesh Agricultural University, Mymensingh, Bangladesh

[*]Corresponding author: md.m.rahman@ndsu.edu; phone: (701) 231-5768; fax: 701-231-8474

[¶]These authors have contributed equally to this work and share first authorship

Abstract

Estimation of genetic diversity in rapeseed/canola is important for sustainable breeding program to provide an option for the development of new breeding lines. The objective of this study was to elucidate the patterns of genetic diversity within and among different structural groups, and measure the extent of linkage disequilibrium (LD) of 383 globally distributed rapeseed/canola germplasm using 8,502 single nucleotide polymorphism (SNP) markers. The germplasm accessions were divided into five subpopulations (P1 to P5) with obvious geographic and growth habit-related patterns. All subpopulations showed moderate genetic diversity (average $H = 0.22$ and $I = 0.34$). The pairwise F_{st} comparison revealed a great degree of divergence ($F_{st} > 0.24$) between most of the combinations. The rutabaga type showed highest divergence with spring and winter types. Higher divergence was also found between winter and spring types. Overall, mean linkage disequilibrium was 0.03 and it decayed to its half maximum within < 45 kb distance for whole genome. The LD decay was slower in C genome (< 93 kb), relative to the A genome (< 21 kb) which was confirmed by availability of larger haplotype blocks in C genome than A genome. To maximize genetic gain, hybridization between rutabaga and other types are potentially the best option. Hybridization between spring and winter, semi-winter type is also helpful to maximize the diversity in subsequent populations. Low genetic differentiation between both spring type subpopulations (P4 and P3) will accelerate favorable allele accumulation for specific traits in elite lines. The Neighbor-Joining tree and kinship matrix will assist to identify distantly related genotypes from subpopulations to utilize in hybridization. The low levels of LD and population structure make the core collection an important resource for association mapping efforts to identify genes useful in crop improvement as well as for selection of parents for hybrid breeding.

Introduction

Rapeseed/canola (*Brassica napus L.*, AACC, 2n = 4x = 38), is a recent allopolyploid of polyphyletic origin that evolved from hybridization events between two parental ancestors of *B. oleracea* (Mediterranean cabbage, CC, 2n = 2x = 18) and *B. rapa* (Asian cabbage or ~~rapeseed~~, AA, 2n = 2x = 20) (1). Rapeseed/canola is the second largest oilseed crops produced in the world after soybean (2). Canola oil is mostly used in frying and baking, margarine, salad dressings, and many other products. Because of its fatty acid profile and the lowest amount of saturated fat among all other oils, it is commonly consumed all over the world and is considered a very healthy oil (3). Canola oil is also rich with alpha-linolenic acid (ALA), which is associated to a lower risk of cardiovascular disease (3). Additionally, canola is utilized as a livestock meal and is the second largest protein meal in the world after soybean (4). In the United States of America, the canola production increased 13.5 folds from five years average of 1991-1995 (0.11 m tons) to five years average of 2015-2019 (1.49 m tons) (5). Due to the growing importance of canola there is a constant need to improve its yield which can be negatively affected by biotic and abiotic stresses. Canola expresses three growth habits, winter, spring and semi-winter. The spring canola is planted in the early spring and harvested in the late spring of the same growing season (6). The winter type canola is seeded in the fall, vernalized over the winter to induce flower and harvested in the summer (6). The semi- winter type is needed for a shorter period of vernalization to induce flower (7). In order to adapt to different growing regions, plants developed systems that sense temperature, light quality, day length, as well as stress signals (8–11). Plants from colder zones require vernalization to flowering (12). Also day length and light quality affect the ability to flower after winter (13). The transition from the vegetative to flowering stage is also controlled by plant hormones and the circadian clock (14, 15). Multiple

studies suggest the Arabidopsis flowering-time gene network might be similar to the one in Brassica (16, 17). Detailed understanding of the nature of genes involved in the growth habits of canola will facilitate development of cultivars adapted to different latitudes (18).

Due to limited use of diversified germplasm in breeding program, development of superior cultivars through traditional breeding might become unsuccessful and lead to stagnation in plant improvement (19). The recent origin of *B. napus* as a species and its very recent domestication (400 years ago), as well as selection on few phenotypes (e.g. low erucic and glucosinolate acids, seed yield) contributed to the low diversity which threatens sustainable production of the crop (20). The narrow genetic diversity might also limit the prospects for hybrid breeding where complementing genepools are needed for the optimal exploitation of heterosis (21). Therefore, it is crucial to study, preserve, and even introduce genetic diversity into rapeseed since the diversity is the best source of biotic and abiotic stress resistance, and various agronomical and morphological traits. Canola improvement can benefit from the availability and detailed characterization of genetically diverse germplasm. The knowledge of population structure, genetic relatedness, and patterns of linkage disequilibrium (LD) are also prime requirements for genome-wide association study (GWAS) and genome selection directed breeding strategies (22, 23).

Multiple genetic diversity and population structure studies, based on LD, have already provided information in regards to genetic diversity in various *B. napus* collections around the world (24 –27). Unfortunately, there is a limited number of studies investigating the genetic variation of canola germplasms in the U.S. core collection justifying a great need for such a research focus. The LD analysis provides an important insight into the history of the species. It also provides valuable direction to breeders in need to diversify their crop gene pools (28). Here

we report a study revealing population structure and LD pattern of the [illegible]S. core collection with good representation of the genetic diversity present in global rapeseed/canola germplasm accessions.

So is it specific to US-adapted germplasm, or does the analysis aim at characterizing the genetic diversity present at the whole world scale ? What are author's objective concerning flowering time data ?. It may be helpful to better define the question addressed in this paper and to prioritize the questions and to present the global strategy proposed by the authors.
It is not clear until the discussion... We need more information in the introduction about the specificity of the US canola breeding programs and the genetic diversity used

Materials and methods

Plant samples and phenotyping

change canola for rapeseed as winter type are not considered as canola

A collection of 383 canola germplasm accessions originated in 24 countries comprising spring, winter, semi-winter, and rutabaga types, were collected from North Central Regional Plant Introduction Station (NCRPIS), Ames, Iowa, USA and North Dakota State University (NDSU) (S1 Table). The collection consists of 156 spring, 152 winter, 58 semi-winter, and 17 rutabaga types. ~~Growth habit was determined by growing the accessions in a greenhouse for at least two seasons and in a field for two years at two locations. Field flowering time was recorded as number of days from seeding date to flowering date where first flowers opened on half of the plants belonging to a single accession. Spring type accessions flowered within 40-60 days after planting, the semi-winter types flowered from 70 to 110 days. On the other hand, winter types did not flower under field conditions. Vernalization treatment was conducted on greenhouse grown winter type accessions to induce flowering and also to confirm the winter habit type according to Rahman and McClean (2013) (6).~~

When was the sowing carried out ? in autumn and spring depending on the genetic material, or in spring for all accessions ? With a spring sowing it was expected that winter accessions were not gowing to flower right ?
need more information on cultural practices and environment characterization (meteorological data, etc...)
Indeed results are not presented => please delete this section

Genotyping and sequencing

Young leaves were collected from 30 days old plants and flash-frozen in liquid nitrogen. Tubes were stored at -80°C until lyophilized. The lyophilized leaf tissue was ground in tubes

with stainless beads using a plate shaker. DNA was extracted using Qiagen DNeasy Kit (Qiagen, CA, USA) from lyophilized tissue following the manufacturer's protocol. DNA concentration was measured using a NanoDrop 2000/2000c Spectrophotometer (Thermofisher Scientific). The ApekI enzyme was used for GBS library preparation (29). Sequencing of the library was done at the University of Texas Southwestern Medical Center, Dallas, Texas, USA using Illumina HiSeq 2500 sequencer.

SNP calling

TASSEL 5 GBSv2 pipeline (30) was used for SNP calling using a 120-base kmer length and minimum kmer count of ten. The reads were aligned to the canola reference genome (31) (available at:

did you also used a winter type reference genome (or a pangenome) as half of the collection was constitued of winter type ?

ftp.ncbi.nlm.nih.gov/genomes/all/GCF/000/686/985/GCF_000686985.2_Bra_napus_v2.0/) using Bowtie 2 (version 2.3.0) alignment tool (32). After passing all the required steps of TASSEL 5 GBSv2 pipeline, 497336 unfiltered SNPs were identified. Then VCFtools (33) was used to select bi-allelic SNPs considering the criteria: minor allele frequency (MAF) ≥ 0.05, missing values (max-missing) ≤50%, depth (minDP) ≥ 5 and physical distance (thin) ≤ 1000 bp. The SNPs that were located outside chromosomes (i.e. position unknown), were removed. As canola is a self-pollinating crop, the SNPs that were heterozygous in more than 25% of total genotypes, were also removed using TASSEL (34). All these filtering steps resulted in a total of 8,502 SNP markers.

This results seems very low, do you have an explanation ?
especially considering the high level of genetic diversity present in the considered germplasm

Data analysis

Need to organize this paragraph according to the main type of analysis performed

The core collection was divided into genetic groups using STRUCTURE v2.3.4 (35) software. The admixture model, a burnin period of 10000 and 50000 Monte Carlo Markov Chain (MCMC) iterations with 10 replications per K (K1-K10), were used as parameters for structure analysis. The optimal number of groups was determined based on DeltaK approach (36) which was performed by Structure Harvester (37). The individual Q matrix for the optimal K value was generated utilizing membership coefficient matrices of ten replicates from STRUCTURE analysis using CLUMPP (38). The results of structure analysis was visualized using the Structure Plot v2 software (39). Principal component analysis (PCA) was conducted by covariance standardized approach in TASSEL (34). An unrooted neighbor-joining (NJ) phylogenetic tree was constructed using MEGAX program with 1000 bootstraps (40). Resulting tree was displayed using FigTree V1.4.4 (41).

Analysis of molecular variance (AMOVA) was done to partition the genetic variance among the groups identified by STRUCTURE in Arlequin3.5. The average pairwise between-population F_{st} values were also calculated using Arlequin3.5 (42). GenAlex v6.5 (43) was used to estimate percentage of polymorphic loci, number of effective alleles, Shannon's information index, expected heterozygosity and unbiased expected heterozygosity of each marker and subpopulation. The SNP distribution plot was developed using R package CMplot (available at: https://github.com/YinLiLin/R-CMplot). The polymorphism information content (PIC) of markers was calculated using software Cervus (44). Tajima's D value of each group was calculated using MEGAX software (40). The kinship (IBS) matrix was calculated using software Numericware i (45), kinship heatmap and histogram were developed using R package

ComplexHeatmap (46). The level of relatedness (IBS coefficients) was correlated with Shannon's information index (I) and diversity (H) in R v3.5.2 (47). Linkage disequilibrium (LD) pattern of whole collection and different subpopulations were analyzed using PopLDdecay (48). The mean linked LD was calculated by dividing total r^2 value with total number of corresponding loci pair. In this case, $r^2 < 0.2$ was considered only. Same procedure was followed to calculate mean unlinked LD where $r^2 \geq 0.2$ was considered. Haplotype block analysis was done using PLINK (49) with a window size of 5 Mb. Confidence interval (CI) method (50) was used to identify haplotype blocks with high LD. Haplotype blocks (>19 Kb), observed in one subpopulation but not in the other, were considered to be subpopulation-specific block. Haplotype blocks (>19 Kb) shared by more than one subpopulation, were considered to be common to corresponding subpopulations.

Are all the analysis usefull to adress the questions raised by authors ? It seems that all existing methods have been tried, but without a clear view of which questions are adressed by each proposed analysis

No analysis of field and greenhouse data ?

Results

SNP profile

The selected 8,502 SNPs were distributed across 19 chromosomes with an average marker density of 1 per 99.5 kb. Chromosome A3 and A4 contained highest (685 SNPs, 8.06%) and lowest (236 SNPs, 2.78%) number of SNPs, respectively. The SNP density was highest on chromosome A7 (71.1 kb) and was lowest on chromosome C9 (134.5 kb) (Table 1, Fig 1). The occurrence of transition SNPs (4,956 SNPs) was more than that of transversions (3,546 SNPs) with a ratio of 1.40. The ratio of transitions to transversions SNPs was higher in A genome (1.41) than that of in C genome (1.38). In both genome, G/C transversions were lowest (4.33% and 4.29%), but A/G and C/T transitions occurred in almost similar frequencies (Table 2). The inbreeding coefficient within individuals (F_{st}), inbreeding coefficient within subpopulations (F_{st}),

Fit? Fis ?

Does these indexes really correspond to Fst??

observed heterozygosity (*Ho*) and fixation index (*F*) of all the markers ranged from -0.45 to 1.00, 0 to 0.73, 0 to 0.57 and 0.40 to 1.00, respectively. The Shannon's information index (*I*) of all markers ranged from 0.10 to 0.69 with a mean value of 0.37. The expected heterozygosity (*He*) ranged from 0.05 to 0.50 with a mean value of 0.27. The polymorphic information content (*PIC*) ranged from 0.05 to 0.37 with a mean value of 0.22 (S2 Table). Subpopulation-wise marker diversity parameters are presented in supplementary S3 Table.

Here, subpopulations are not described yet,
So it remains difficult to interpret values for
subpopulation characterization.

Table 1. Chromosomewise distribution of SNP markers.

Chromosome	No. of SNPs	% SNPs	Start position	End position	Length (Mb)	Density (Kb)
A1	440	5.18	149163	35806075	35.7	81.0
A2	392	4.61	13430	34692905	34.7	88.5
A3	685	8.06	2769	49103583	49.1	71.7
A4	236	2.78	32805	23517671	23.5	99.5
A5	413	4.86	18668	31435105	31.4	76.1
A6	448	5.27	120409	36005103	35.9	80.1
A7	384	4.52	85869	27388322	27.3	71.1
A8	281	3.31	231427	27734410	27.5	97.9
A9	541	6.36	81404	45841268	45.8	84.6
A10	305	3.59	133853	22085737	22.0	72.0
C1	445	5.23	86671	50660872	50.6	113.7
C2	589	6.93	92431	68260222	68.2	115.7
C3	651	7.66	3839	80365889	80.36	123.4
C4	634	7.46	138930	70507417	70.4	111.0
C5	366	4.30	26760	44124497	44.1	120.5
C6	414	4.87	275190	45479327	45.2	109.2
C7	518	6.09	271113	62304827	62.0	119.8
C8	383	4.50	57934	46317429	46.3	120.8
C9	377	4.43	920885	51627086	50.7	134.5
Mean	447.47					99.5

to what correspond start and end position ? to the chromosome length compared to the reference genome ?
Density ? length divided by SNP number ?

Fig 1. Chromosome-wise SNP density map. Frequency of SNPs varies according to color gradient.

Table 2. Transition and transversion SNPs across the genome.

Genome	SNP type	Model	No. of sites	Frequencies (%)	Total (percentage)
A	Transitions	A/G	1195	14.06	2416 (28.3%)
		C/T	1221	14.36	
	Transversions	A/T	457	5.38	1709 (20.1%)
		A/C	424	4.99	
		G/T	460	5.41	
		G/C	368	4.33	
C	Transitions	A/G	1273	14.97	2540 (29.9%)
		C/T	1267	14.90	
	Transversions	A/T	496	5.83	1837(21.6%)
		A/C	482	5.67	
		G/T	494	5.81	
		G/C	365	4.29	

Population structure

The whole collection was divided into six subpopulations based on structure analysis using the Delta K approach (Fig 2A). Genotypes of different types and origins were well clustered. The winter, semi-winter, rutabaga type genotypes were grouped under subpopulation-1 (P1), subpopulation-2 (P2), and subpopulation-5 (P5), respectively. The spring type genotypes were grouped under two subpopulations: subpopulation-3 (P3) and subppulation-4 (P4) whereas subpopulation-6 (P6) composed of only nine genotypes of different types (Fig 2B). Though each subpopulation consisted of genotypes of different origin, P1, P2 and P4 were dominated by European, Asian and American (NDSU breeding lines) genotypes, respectively, whereas P3, P5 and P6 were composed of genotypes of mixed origin. We performed principal component analysis (PCA) to show the genetic similarity among subpopulations and genotypes. The first two axes explained 21% of the total observed variation (S4 Table). The PCA revealed that subpopulation P1, P2, P3, P4 and P5 were well clustered and separated from each other, but genotypes of P6 were scattered within other subpopulations (Fig 3). In addition to that, we also

constructed phylogenetic tree based on neighbor joining (NJ) criteria (Fig 4). The output of neighbor-joining (NJ) tree analysis was in line with that of PCA. Based on the PCA and NJ output, we merged the P6 genotypes into P1, P2, P3 and P4 and considered five subpopulations for further analysis and discussions. Based on individual Q matrix, the proportion of pure (~~non-hybrid~~) and admixed (containing genotypes ?, the markers are not assigned to subpopulation ~~markers~~ assigned to more than one subpopulation) genotypes in each subpopulation was calculated. The proportion of ~~pure~~ accessions in subpopulations ranged from 41% to 80% at a 0.7 cutoff value and 10% to 35% at 0.9 cutoff value (Table 3).

pure line vs hybrid is different from non-admixed vs admixed, a pure line can be admixed

Fig 2. Bayesian clustering of whole collection using 8,502 SNP markers in STRUCTURE v. 2.3.4. (A) Graphical representation of optimal number of clusters (K) determined by Evanno's method, where highest Delta K indicates the number of subpopulations. (B) Estimated population structure (P1 to P6) of 383 ~~canola~~ rapeseed genotypes on K=6 according to Delta K.

Fig 3. Principal component analysis of SNP diversity based on genetic distance. Colors represent different subpopulations identified at K = 6 in figure 2. Genotypes belong to P6 (black dots) are scattered within different subpopulations.

Fig 4. Phylogenetic tree based on neighbor-joining (NJ) using information from 8,502 SNP markers. Each branch is color-coded according to membership into the K= 6 subpopulations identified by structure in figure 2. Subpopulations bear same color as in figure 3.

Table 3. Number of ~~pure and~~ admixed individuals per subpopulation. Need to better explain to what correspond the cutoff threshold.

eg if more than 70% of the genome of an accession is attributed to the same subpopulation, then the accession is considered as non-admixed..

Subpopulations	Total no. of genotypes	0.7 cutoff		0.9 cutoff	
		No. of admixed ? genotypes	% of from total	No. of genotypes	% of from total
P1	152	89	58.6	42	27.6
P2	52	24	46.2	9	17.3
P3	98	40	40.8	14	14.3
P4	61	30	49.2	6	9.8
P5	20	16	80.0	7	35.0
Total	383	199	52.0	78	20.4

Population diversity

In all subpopulations, the percentage of polymorphic loci was greater than 75%. It was highest in P1 (99%) and lowest in P5 (75%). The diversity (*H*) of the five subpopulations ranged from 0.19 (P4 and P5) to 0.25 (P2) with an average of 0.22. The Shannon's information index (*I*) ranged from 0.31 (P4 and P5) to 0.40 (P2) with an average of 0.34. The Tajima's D value ranged from -0.70 (P4) to 0.53 (P1) with an average of 0.13 (Table 4).

Table 4. Subpopulation-wise diversity parameters.

Subpopulations	Polymorphic loci (%)	*Na* [a]	*Ne* [b]	*I* [c]	*H* [d]	*Uh* [e]	Tajima's D*
P1	99.12	1.99	1.32	0.35	0.21	0.21	0.53
P2	94.32	1.94	1.40	0.40	0.25	0.25	0.30
P3	96.98	1.97	1.35	0.36	0.22	0.23	0.30
P4	80.67	1.81	1.30	0.31	0.19	0.19	-0.70
P5	75.25	1.75	1.31	0.31	0.19	0.20	0.23
Mean	89.27	1.89	1.34	0.34	0.22	0.22	0.13

[a] No. of different alleles, [b] No. of effective alleles, [c] Shannon's information index [d] Diversity, [e] Unbiased diversity. SE (standard error) was zero in all cases. Indices calculated using 8191 SNPs with GenAlex v. 6.5. * was calculated with 1000 permutations.

Population genetic differentiation

The analysis of molecular variance (AMOVA) revealed that variance among subpopulations covered 24% of total variation whereas the remaining 76% of total variation accounted for variance among individuals within subpopulations (Table 5) with a F_{st} and *Nm* value of 0.24 and 1.28, respectively.

Table 5. Summary of AMOVA.

I don't understand the number of df for the withsubpopulation. The population was constitued of 383 genotypes so a total of 382 df, 4 for controlling the subpopulations, we expect 378 for the within subpopulations effect no ?

Sources of variation	d.f.	Sum of squares	Variance components	% of variation	F_{st}	Nm
Among subpopulations	4	130814.6	228.5***	23.5	0.24	1.28
Within subpopulations	761	565699.6	743.4	76.5		
Total	765	696514.1	971.9			

ok, in fact, you consider two observations per genotype (diploid).
I was wondering if it was ok if all accessions consist in pure line., did you present the mean level of heterozygoty per accession ?

*** indicates $p < 0.001$ for 1023 permutations

All pairwise F_{st} comparisons between subpopulations were significant ($p < 0.01$). All combinations showed a great degree of divergence ($F_{st} > 0.20$) (Wright 1943), except combinations P3 and P4 (0.11), P1 and P2 (0.19). The pairwise $F_{st} > 0.30$ was observed between P1 and P5, P3 and P5, P4 and P5 (Table 6).

Table 6. Genetic differentiation among subpopulations.

	Subpopulation pairwise *Fst*				
	P1	P2	P3	P4	P5
P1	0				
P2	0.19**	0			
P3	0.25**	0.24**	0		
P4	0.21**	0.24**	0.11**	0	
P5	0.34**	0.24**	0.34**	0.39**	0

Diagonal values are pairwise Fst comparisons, performing 1000 permutations using Arlequin v. 3.5. **indicates $p < 0.01$

We performed kinship (IBS) analysis to facilitate the individual genotype selection for desirable cross combinations (Fig 5, S5 Table). In whole collection, the IBS coefficients ranged from 1.21 to 1.94. The average coancestry between any two canola genotypes was 1.47. The P2 contained almost 50% of total genotypic pairs having IBS coefficients less than 1.50 whereas this portion was comparatively lower in other subpopulations (Table 7, S1 Fig).

Fig 5. Heatmap of kinship matrix of entire collection.

Table 7. Summary of subpopulation-wise kinship (IBS) matrix.

Subpopulations	Whole collection	P1	P2	P3	P4	P5
IBS coefficients range	1.21- 1.94	1.40- 1.94	1.27- 1.93	1.29- 1.93	1.46-1.94	1.35-1.92
Mean of IBS coefficients	1.47	1.58	1.49	1.55	1.62	1.60
Pairs having ≤ 1.50 IBS coefficients (%)	63.9	9.6	50.7	21.7	1.1	18.0
Pairs having > 1.50 IBS coefficients (%)	36.1	90.4	49.3	78.3	98.9	82.0

We also performed correlation analysis between mean pairwise relatedness (IBS coefficients) among individuals within subpopulation and Shannon's information index (*I*), diversity (*H*). The *I* and *H* were significantly and negatively correlated with relatedness (r= -0.97, -0.98, and p<0.01), respectively.

Linkage disequilibrium pattern

The linkage disequilibrium (LD) pattern was investigated across the entire collection, each subpopulation, genome, and chromosome-wise. LD = r^2 values decreased with the increase of distances. In all cases, mean LD was high (r^2 > 0.22) at short distance bin (0-2 kb) and declined with increasing bin distance (S6 Table). In the entire collection considering both A and C genome, the mean linked LD, mean unlinked LD and loci pair under linked LD was 0.44, 0.02 and 1.81%, respectively. The mean linked LD was similar in P3 and P4 (r^2 = 0.45), lower in P2

please reformulate. What do you define as "linked LD". LD stands for linkage disequilibrium, then linked and in "LD" may be synonymous ?

(r^2= 0.41) and higher in P1 (r^2= 0.48). The loci pair in linked LD was higher in P5 (8.76%) and lowest in P1 (1.52%). The mean linked LD, mean LD and loci pair under linked LD was always higher in all cases in case of C genome than that of A genome (Table 8). We also calculated the LD decay rate. In the whole collection, LD decayed to its half maximum within < 45 kb distance for whole genome, < 21 kb for A genome, and < 93 kb for C genome. In all subpopulations, the distance for LD decay to its half maximum was always higher for C genome than A genome. Each chromosome showed differential rate of LD decay (S2 and S3 Fig). LD persisted the longest in chromosome C1 (348 kb) and C2 (244 kb). The decay distance was shortest in chromosome A5 (13 kb) and A1 (16 kb) (S6 Table). LD decayed to its half –maximum within < 29 kb for P1, <45 kb for P2, P3, <101 kb for P4, and <120 kb for P5. In all subpopulations LD persisted also longest in all chromosomes of C genome than that of A genome (Fig 6, S7 Table).

please reformulate

???

what is a half maximum ?

why not considering a common LD threshold to estimate the LD decay ?

Can you compare the LD decay as the chosen LD threshold is different between chromosome ?

Fig 6. Linkage disequilibrium (LD) differences and decay pattern among subpopulations.

Table 8. Linkage disequilibrium in the studied collection.

please clarify everywhere in the manuscript the notion of Linked LD and unlinked LD

Subpopulation	Mean ??? linked LD	Mean ???? unlinked LD	Mean LD	Loci pairs in linked LD (%)	Loci pairs in unlinked LD (%)
			AC_Genome		
Whole collection	0.44	0.02	0.03	1.81	98.2
P1	0.48	0.01	0.02	1.52	98.5
P2	0.41	0.02	0.03	2.65	97.4
P3	0.45	0.02	0.03	1.94	98.1
P4	0.45	0.02	0.04	3.98	96.0
P5	0.43	0.03	0.07	8.76	91.2
			A_Genome		
Whole collection	0.33	0.02	0.02	1.34	98.7
P1	0.38	0.01	0.02	1.12	98.9
P2	0.32	0.02	0.03	2.02	98.0
P3	0.36	0.02	0.02	1.41	98.6
P4	0.40	0.02	0.03	3.45	96.6
P5	0.38	0.04	0.06	7.06	92.9
			C_Genome		
Whole collection	0.50	0.02	0.03	2.21	97.8
P1	0.52	0.01	0.02	1.83	98.2
P2	0.46	0.02	0.04	3.27	96.7
P3	0.50	0.02	0.03	2.35	97.7
P4	0.48	0.02	0.04	4.41	95.6
P5	0.46	0.03	0.08	10.57	89.4

We also performed haplotype block analysis to investigate LD variation patterns across whole genome. A total 200 blocks covering 18 Mb out of the 976 Mb anchored *B. napus* reference genome (31), were identified. A and C genome contained 67 and 133 haplotype blocks, respectively. The total length of A and C genome specific haplotype blocks were 1.8 Mb and 16 Mb, respectively. The total length of haplotype blocks varied greatly from chromosome to chromosome. Total haplotype block length varies from 24 Kb on A1 to 901 Kb on A9 in A genome and in C genome it varies between 40 Kb on C9 to 3,610 Kb on C2 (Table 9). The subpopulation-specific and common haplotype blocks were shown in supplementary S8 Table.

considering the aim of the study, these results may be presented in the manuscript and not as supp data, do you see any differences in haplotypes between subpopulations ?

Table 9. Subpopulation-wise number and length of haplotype blocks (HBs) along chromosomes.

???

how can you have more haplotype in single subpopulations (P1 or P3) than at the entire panel scale ?

	Entire panel		P1		P2		P3		P4		P5	
Chr.	No[a]	Size[b]	No[a]	Size[b]	No[a]	Size[b]	No[a]	Size[b]	No[a]	Size [b]	No[a]	Size[b]
A1	5	24	6	733	2	5	6	557	5	2080	0	0
A2	8	80	7	96	1	11	8	654	3	15	0	0
A3	6	46	5	29	5	44	8	51	10	927	1	8
A4	5	46	1	27	2	17	0	0	1	6	0	0
A5	7	57	2	13	3	503	5	138	5	427	1	1
A6	9	308	6	564	4	29	5	52	5	51	0	0
A7	8	70	7	72	2	15	7	412	9	1092	1	13
A8	4	304	1	2	3	21	2	234	6	2654	0	0
A9	10	901	7	62	14	1493	6	64	9	1393	1	21
A10	5	29	6	50	2	21	1	6	5	45	1	1
C1	23	3099	14	3314	14	4638	19	2947	20	4295	1	14
C2	26	3611	19	3141	9	4503	22	3756	16	5237	7	3594
C3	14	969	13	930	13	1480	16	1603	9	1070	2	192
C4	16	3440	15	5351	15	5330	14	4502	13	6063	4	3989
C5	9	423	8	410	10	516	7	1148	7	2679	1	2
C6	18	1204	9	972	7	1191	13	1206	15	3357	3	954
C7	17	2394	17	2583	7	70	16	2479	10	1414	1	13
C8	4	893	5	865	10	1289	6	941	7	1143	1	73
C9	6	41	7	49	4	438	7	223	3	11	1	14
AC Genome	200	17938	155	19264	127	21615	168	20975	158	33956	26	8888
A Genome	67	1865	48	1648	38	2160	48	2168	58	8688	5	43
C Genome	133	16073	107	17616	89	19455	120	18807	100	25267	21	8845

[a] The number of haplotype blocks on each chromosome.

[b] The total length of haplotype blocks for each chromosome in Kb.

Discussion

Genotyping-by-sequencing (29) is one approach to obtain high frequency SNPs. The strategy has been used for population genetic studies, association mapping, and proven to be a powerful tool to dissect multiple genes/QTL in many plant species (51–53). We obtained 497,336 unfiltered SNPs markers of which 8,502 high quality SNP marker were used for genetic

diversity analysis of 383 genotypes. Delourme et al. (2013) (24) conducted genetic diversity analysis in *B. napus* using 7,367 SNP markers of 374 genotypes. However, different marker technologies such as Single Sequence Repeat (SSR), Sequence Related Amplified Polymorphism (SRAP) markers have been used by other researchers for genetic diversity analysis in *B. napus*. Chen et al. (2020) (54) used 30 SSR markers, Wu et al. (2014) (55) utilized 45 SSR markers, Ahmad et al. (2014) (56) used 20 SRAP markers for genetic diversity and population structure analysis of *B. napus*. Earlier, our group conducted a genetic diversity study of flax using 373 germplasm accessions with 6,200 SNP markers.

The SNP markers were distributed throughout 19 chromosomes of *B. napus* and the marker density was 1 per 99.5 Kbp. This is comparable density to earlier study conducted by Delourme et al. (2013) (24). Therefore, this marker density provides a sufficient resolution to accurately estimate genome-wide diversity as well as the extent of LD within the genome. The high marker density will also help association mapping studies precisely identify a causal locus/loci or very closely linked loci that can be further used either in MAS or to pinpoint the causative locus (57).

No, the SNP resolution is not sufficient to target causative locus in rapeseed, especially for seed yield which is highly polygenic.

The core collection utilized in this study represents mostly adapted lines from various breeding programs rather than wild accessions commonly used in diversity studies. Therefore, sources of variation, markers of interest identified in the collection can be directly used in breeding programs.

The authors did not provide any information on how the core collection was defined. A core collection is supposed the represent the maximum diversity of an initial collection within a small number of accessions. Here, they gathered 383 accessions. So I won't consider it as a core collection.

We have identified higher frequency of transition SNPs over transversion SNPs that is an agreement with Bus et al. (2012) (58), Clarke et al. (2013) (59), and Huang et al. (2013) (60) in *B. napus*. Higher number of transition SNPs over transversion is also reported in other crop species such as *Hevea brasiliensis* (61), *Camellia sinensis* (62), *Camelina sativa* (63), and *Linum*

usitatissimum (64). The high number of transition SNPs indicating that this mutation is more tolerable to natural selection (65).

We have calculated the polymorphic information content (PIC) and expected heterozygosity (*He*) for each marker. The PIC indicates the usefulness of any marker for linkage analysis, and *He* determines the diversity of haploid markers (66). In our research, the PIC value is ranged from 0.05 to 0.35 indicating that the markers are moderate or low informative. The similar lower PIC value (0.1 to 0.35) was reported by Delourme et al. (2013) (24) in *B. napus*. The lower PIC value was also reported in winter wheat (67), maize (68), flax (64, 69) and rice (70). The lower PIC value is a result of bi-allelic nature of SNP markers and probable low mutation rate (71). In our study, the *He* value of each marker was always greater than corresponding PIC value indicating an average lower allele frequency in our population (66).

Population structure and diversity

Multiple population-based analyses, including population structure, PCA and NJ tree analysis split the core collection into five distinct clusters. Previously, three clusters have been reported based on winter, spring and semi-winter growth habit types of *B. napus* (20, 24–26,72–74). Our analysis showed a clear trend of the five clusters belonging to winter type (P1), semi-winter type (P2), spring type of mixed origin (P3), spring type developed at NDSU (P4), and rutabaga type (P5) growth habits. Our finding showed strong agreement with other studies to separate winter, semi-winter and spring types. Moreover, we could separate the rutabaga types and divided the spring type into two groups: American origin and the rest of other countries origin. This additional cluster added new information to select germplasm from different subpopulation for increasing genetic diversity in canola breeding program.

We have identified a moderate diversity (average *H* = 0.22) within the subpopulations. *B. napus* is capable of self-pollination, and little cross pollination may be occurred by insect. Being a mostly self-pollinated crop a low to moderate subpopulation diversity in *B. napus* is expected. Low to moderate diversity was also found in previous studies (75–77). Along with the reproduction system, one needs to look at evolution and domestication history for explaining low to moderate levels of diversity in *B. napus*. This allopolyploid species originated at Mediterranean coast as a result of a natural cross between *B. rapa* and *B. oleracea* which occurred approximately 0.12 -1.37 million years ago (78, 79). The domestication of *B. napus* occurred very recently, around 400 years ago with the first rapeseed being most likely a semi-winter type due to the mild climate in the region (72, 80). Later on, European growers developed the winter and spring type Brassicas through selection for cold hardiness or early flowering to expand its cultivation in further North in the last century (81). Therefore, the low to moderate diversity in winter and spring *B. napus* can be mostly explained by a recent history of the species, followed by infrequent exchange of genetic material with other Brassicas (24), as well as by the traditional breeding practices selecting for only few phenotypes. In our study, the more diversity in semi-winter type (P2, *H*= 0.25) than winter (P1, *H*= 0.21) and spring (P4, *H*= 0.19) type is supported by its domestication history. We have seen a homogeneity of the diversity indices of different subpopulations indicating that the species is stable enough to avoid the natural loss of genetic variability by genetic drift (82). The *Nm* value was greater than one, which indicates that there was enough gene flow among semi-winter, winter and spring types. These findings also support the evolution of winter and spring types from semi-winter type. In this research, Tajima's D value was calculated to identify different subpopulations with availability or scarcity of rare alleles (83). P4 (spring type originated at NDSU, USA) showed a negative

How ?

Tajima's D value indicating presence of more rare and unique alleles and recent expansion of this subpopulation compared to the other subpopulations (84). Recently, the NDSU canola breeding program developed the P4 advanced breeding lines through crossing different genetic resources including winter, spring and semi winter types and subsequent selection. Therefore, the P4 displayed an abundance of rare alleles. The subpopulation P1, P2, P3, and P5 showed positive Tajima's D value indicating an excess of intermediate frequency alleles, which may be caused by balancing selection, population bottleneck, or population subdivision. Previously, negative Tajima's D values were found in spring and winter type B. napus accessions (74). The negative correlation between diversity indices (*H* and *I*) and relatedness (average IBS coefficients) indicates that inbreeding and genetic drift play a significant role in reducing genetic variability in the studied population which results in increased differentiation among sub-populations. Similar phenomenon was also found in flax (64) and *Arapaima gigas* species (85).

This information should have been given since the very beginning of the manuscript

Parent selection for crossing from diverged population will allow us to create a new population with increased genetic diversity and transgressive segregation, and eventually will increase genetic gain. Pairwise F_{st} statistic, a parameter describing population structure differentiation and the degree of divergence among populations (86), was estimated among five subpopulations. In the present study all pairwise F_{st} values comprising both low and high values, were statistically significant. Similar type results were also found in other studies (74, 87–89). Lower pairwise F_{st} (0.11) was identified between spring type originated in USA (P4) and spring type originated in other countries (P3). This is reasonably justified as both subpopulations comprise of spring type genotypes and germplasm exchanged occurred between USA and other countries. It also indicating that we will not get higher genetic diversity in population if we use only spring types in the crossing program. But this combination is good for accumulating

specific elite trait if the targeted trait is found in members of one and missing from the members of another group. We found spring type (P3 and P4) genotypes are greatly divergent ($F_{st} > 0.20$) from winter and semi-winter type (P1 and P2) genotypes. Utilization of genotypes from these group in crossing program will broaden the genetic base of developed population results in high heterosis. This potentiality has already been proved as hybrids between the Chinese semi-winter and European (including Canada) spring type exhibited high heterosis for seed yield (90). The P5 (rutabaga type) showed the higher F_{st} with other subpopulations such as the highest F_{st} was observed between P5 and P4 (NDSU spring type) followed by P3 (other spring type), P1 (winter type) and P2 (semi-winter type). This outcome clearly shows that rutabaga is genetically distinct from spring and winter type canola which is confirmed by previous studies (72, 73, 91). This distinctness of rutabaga can be exploited through heterosis breeding. Several previous studies have already showed rutabaga as a potential gene pool for the improvement of spring canola (92, 93). NDSU canola breeding program also utilized winter and rutabaga types in the breeding program for increasing genetic diversity and for improvement of spring canola.

Crossing among genotypes within subpopulations is also useful as variation among individuals within subpopulations is greater than that among subpopulations, which was revealed by AMOVA. This finding is also in agreement with the previous findings (54, 77, 94, 95). In this case subpopulations P2, P3 and P1 showing high diversity ($H > 0.20$) could be utilized for cultivar development. Structure, NJ tree and PC analysis revealed that all subpopulations contained both pure (non-hybrid) as well as admixed (share ~~SNPs~~ alleles from different subpopulation) genotypes. For broadening genetic base of population, pure genotypes should be crossed. But for improving or introgression of specific traits, admixed genotypes could also be crossed which will reduce the population size required for phenotypic screening. However, population diversity

why only pure line ?

indicates the level of similarity or dissimilarity based on alleles that not necessarily come from the same parent or ancestor, this tends to inflate the real differentiation between any two pair of individuals (96). Since a breeder would like to combine positive alleles that historically never have been combined, IBS values are good to decide what individuals will be crossed. Low IBS is

sometimes it is also good to maintain "good combinations" present in elite background, and not shuffle everything by crossing too disctinct genotypes

the best. But, IBS values in self-pollinated crops tends to be higher than that in cross-pollinated crops as heterozygosity reduces the probability of two alleles at a locus of being identical by state (97). In our study, most of the genotypes had weak relatedness as approximately 64% of pairwise coancestry ranged from 1.21 to 1.50. Crossing among genotypes from subpopulation P2 will demonstrate more diversity, than that of other subpopulations, as most genotypic combinations of P2 shows low IBS coefficients than others. This finding is in line with the evolutionary history of B. napus where semi-winter type is the base population containing more divergence. Gradually this diversity is narrowed down in P3 (spring type, mixed origin) and P1 (winter type), because genotypic pairs belong to P3 and P1 having high IBS values evolved from semi-winter type (81). Subpopulation P4 exhibited highest number of pairs having IBS > 1.5, which is obvious as these genotypes are advanced breeding lines developed from crossing of same set of parents in different combinations. Genotypic pairs of P5 (rutabaga type) also showed high coancestry which is may be due to the duplicates. We could discard the duplicates during the crossing program.

Linkage Disequilibrium

From this point onwards, it seems as if someone else wrote the discussion....

Linkage disequilibrium can be defined as the correlation among polymorphisms in a given population (98). The strength of association mapping relies on the degree of LD between the genotyped marker and the functional variant. Linkage disequilibrium analysis provides

insight into the history of both natural and artificial selection (breeding) and can give valuable guidance to breeders seeking to diversify crop gene pools (20). SNPs in strong LD are organized into haplotype blocks which can extend even up to few Mb based on the species and the population used. Genetic variation across the genome is defined by these haplotype blocks. Haplotypes which are subpopulation-specific are defined by various demographic parameters like population structure, domestication, selection in combination with mutation and recombination events. Conserved haplotype structure can then be used for the identification and characterization of functionally important genomic regions during evolution and/or selection (99). Also, the extent of LD needs to be quantified across the genome at high resolution (down to approximately 1 Kbp) (100). The information is important for choosing crossing schemes, association studies and germplasm preservation strategies (101–104).

We used markers from across the genome to quantify the LD for the core collection. Low level of LD was evident for each individual subpopulation in A, C and whole gnome. The low level of LD can be due to multiple factors. First of all, canola is a partially outcrossing species with an average of 21-30 % of cross pollination (105–107). The outcrossing occurring in canola leads to more recombination and to a breakdown of haplotype blocks. Secondly, the ancestral history of canola is limited in comparison with other crops, such as rice, common bean, wheat and corn, restricting the selection of desirable haplotypes during the evolution. In other words, there was no adaptation or domestication pressure on the species, which would lead towards positive selection. Third, the only selection pressure imposed on the species for a relatively short time was breeding. However, the breeding practices were biased towards selection of only few phenotypes. Additionally, the short period of time under selection pressure might have not been sufficient to select favorable haplotypes in the genome. Fourth, since canola cultivars with

?????? (it seems to me that the results previsously prensented were in contradiction ?

different growth habits are compatible there has been always gene flow present between them contributing to the low level of LD. The N_m >1 was observed in this study, which supports this gene flow. Finally, the restriction enzyme used to develop the libraries for sequencing of the core collection helped in identification of SNPs largely residing in genic regions, which are prone to high recombination, contributing to the low level of LD.

In this study, we have identified that the LD decay in B. napus varied across chromosomes of both A and C genomes. In addition, LD in C genome decayed much slower than A genome. C genome also contained larger haplotype blocks than A genome. This LD patterns are consistent with previous findings (20, 27, 108–110). The slower LD decay and presence of long haplotype blocks in C genome indicates that high level of gene conservation could have resulted from limited natural recombination or could be exchanged of large chromosomal segment during evolution. In the whole genome, presence of subpopulation specific haplotype blocks suggests that these regions had been experienced selection pressure for specific geographic regions adaptation. In all subpopulations, presence of shorter haplotype blocks in A genome than C genome reveals that B. rapa progenitor of B. napus containing A genome, which has been used as oilseed crop and probably being used in hybridization process. Sharing haplotype blocks by different subpopulations specially in C genome also confirms its conserved nature. The low level of LD or haplotype blocks has implications for association mapping and a proper experimentation design is necessary for utilizing a reduced set of markers by tagging major haplotypes (111). Though low LD requires more markers to pinpoint the location of various QTL, but once a marker is found to be significantly associated with a phenotype, there might be a higher probability of identifying the casual gene.

Conclusions

This study provided a new insight to select the best parents in crossing plan to maximize genetic gain in the population. The population structure analysis showed a clear geographic and growth habit related clustering. Low LD values indicate that our collection is a valuable resource for prospect association mapping endeavors. The genetic diversity of the core collection of *B. napus* was low. Breeding efforts will need to address this issue in order to generate future hardy and high yielding varieties with resistance to many abiotic and biotic stresses. The rutabaga type showed the highest genetic divergence with spring and winter types accessions. Therefore, the breeding strategies to increase the genetic diversity may include generating population from rutabaga and spring crosses, or using rutabaga and winter crosses.

Acknowledgements

The authors thank Mr. Andrew Ross (Department of Plant Sciences, NDSU, Fargo, ND) for his help in planting and maintaining the plants in the greenhouse and in the field.

References

1. Nagaharu U. Genome-analysis in Brassica with special reference to the experimental formation of *B. napus* and peculiar mode of fertilization. Japanese J Bot. 1935; 7: 389-452.
2. USDA Foreign Agricultural Service. Oilseeds: World markets and trade reports. 2020; https://apps.fas.usda.gov/psdonline/circulars/oilseeds.pdf. [Accessed February 20, 2020].
3. Connor WE. Importance of n-3 fatty acids in health and disease. American Journal of Clinical Nutrition. 2000; 1: 171S-175S. doi:10.1093/ajcn/71.1.171s.
4. Swanepoel N, Robinson PH, Erasmus LJ. Effects of ruminally protected methionine and/or phenylalanine on performance of high producing Holstein cows fed rations with very high levels of canola meal. Anim Feed Sci Technol. 2015; 205:10–22. doi: 10.1016/j.anifeedsci.2015.04.002.
5. NASS. National Agricultural Statistics Service. 2020; https://www.nass.usda.gov. [Accessed February 20, 2020].
6. Rahman M, Mcclean P. Genetic analysis on flowering time and root system in *brassica napus* L. Crop Sci. 2013; 53: 141-147. doi:10.2135/cropsci2012.02.0095.
7. Wang N, Qian W, Suppanz I, Wei L, Mao B, Long Y, et al. Flowering time variation in oilseed rape (Brassica napus L.) is associated with allelic variation in the FRIGIDA homologue BnaA.FRI.a. J Exp Bot. 2011; 62: 5641–5658. doi:10.1093/jxb/err249.
8. Jaeger KE, Graf A, Wigge PA. The control of flowering in time and space. Journal of experimental botany. 2006; 57: 3415–3418. doi:10.1093/jxb/erl159.

9. Jung C, Müller AE. Flowering time control and applications in plant breeding. Trends in Plant Science. 2009; 14: 563–573. doi:10.1016/j.tplants.2009.07.005.

10. Srikanth A, Schmid M. Regulation of flowering time: All roads lead to Rome. Cellular and Molecular Life Sciences. 2011; 68: 2013–2037. doi:10.1007/s00018-011-0673-y.

11. Wigge PA. Ambient temperature signalling in plants. Current Opinion in Plant Biology. 2013; 16: 661–666. doi:10.1016/j.pbi.2013.08.004.

12. Preston JC, Sandve SR. Adaptation to seasonality and the winter freeze. Frontiers in Plant Science. 2013; 4:167. doi:10.3389/fpls.2013.00167.

13. Song J, Irwin J, Dean C. Remembering the prolonged cold of winter. Current Biology. 2013; 23: R807–R811. doi:10.1016/j.cub.2013.07.027.

14. Pak H, Guo Y, Chen M, Chen K, Li Y, Hua S, et al. The effect of exogenous methyl jasmonate on the flowering time, floral organ morphology, and transcript levels of a group of genes implicated in the development of oilseed rape flowers (*Brassica napus* L.). Planta. 2009; 231: 79–91. doi:10.1007/s00425-009-1029-9.

15. de Montaigu A, Tóth R, Coupland G. Plant development goes like clockwork. Trends in Genetics. 2010; 26: 296–306. doi:10.1016/j.tig.2010.04.003.

16. Wang JW, Czech B, Weigel D. miR156-Regulated SPL Transcription Factors Define an Endogenous Flowering Pathway in *Arabidopsis thaliana*. Cell. 2009; 138: 738-749. doi:10.1016/j.cell.2009.06.014.

17. Zou X, Suppanz I, Raman H, Hou J, Wang J, Long Y, et al. Comparative Analysis of FLC Homologues in Brassicaceae Provides Insight into Their Role in the Evolution of Oilseed Rape. PLoS One. 2012; 7: e45751. doi:10.1371/journal.pone.0045751.

18. Kippes N, Debernardi JM, Vasquez-Gross HA, Akpinar BA, Budak H, Kato K, et al. Identification of the VERNALIZATION 4 gene reveals the origin of spring growth habit in ancient wheats from South Asia. Proc Natl Acad Sci U S A. 2015; 112: E5401-E5410. doi:10.1073/pnas.1514883112.

19. Rahman H. Review: Breeding spring canola (Brassica napus L.) by the use of exotic germplasm. Can J Plant Sci. 2013; 93: 363–373. doi:10.4141/CJPS2012-074.

20. Qian L, Qian W, Snowdon RJ. Sub-genomic selection patterns as a signature of breeding in the allopolyploid Brassica napus genome. BMC Genomics. 2014; 15:1170. doi:10.1186/1471-2164-15-1170.

21. Girke A, Schierholt A, Becker HC. Extending the rapeseed genepool with resynthesized Brassica napus L. I: Genetic diversity. Genet Resour Crop Evol. 2012; 59: 1441–1447. doi:10.1007/s10722-011-9772-8.

22. Iqbal MJ, Mamidi S, Ahsan R, Kianian SF, Coyne CJ, Hamama AA, et al. Population structure and linkage disequilibrium in Lupinus albus L. germplasm and its implication for association mapping. Theor Appl Genet. 2012; 125: 517–530. doi:10.1007/s00122-012-1850-6.

23. Gurung S, Mamidi S, Bonman JM, Xiong M, Brown-Guedira G, Adhikari TB. Genome-wide association study reveals novel quantitative trait loci associated with resistance to multiple leaf spot diseases of spring wheat. PLoS One. 2014; 9: e108179. doi:10.1371/journal.pone.0108179.

24. Delourme R, Falentin C, Fomeju BF, Boillot M, Lassalle G, André I, et al. High-density SNP-based genetic map development and linkage disequilibrium assessment in *Brassica napus* L. BMC Genomics. 2013; 14:120. doi:10.1186/1471-2164-14-120.

25. Li F, Chen B, Xu K, Wu J, Song W, Bancroft I, et al. Genome-wide association study dissects the genetic architecture of seed weight and seed quality in rapeseed (*Brassica napus* L.). DNA Res. 2014; 21: 355–367. doi:10.1093/dnares/dsu002.

26. Raman H, Dalton-Morgan J, Diffey S, Raman R, Alamery S, Edwards D, et al. SNP markers-based map construction and genome-wide linkage analysis in *Brassica napus*. Plant Biotechnol J. 2014; 12: 851–860. doi:10.1111/pbi.12186.

27. Wang N, Li F, Chen B, Xu K, Yan G, Qiao J, et al. Genome‐wide investigation of genetic changes during modern breeding of *Brassica napus*. Theor Appl Genet. 2014; 127: 1817–1829. doi:10.1007/s00122-014-2343-6.

28. Voss‐ Fels K, Frisch M, Qian L, Kontowski S, Friedt W, Gottwald S, et al. Subgenomic Diversity Patterns Caused by Directional Selection in Bread Wheat Gene Pools. Plant Genome. 2015; 8: 1-13. doi:10.3835/plantgenome2015.03.0013.

29. Elshire RJ, Glaubitz JC, Sun Q, Poland JA, Kawamoto K, Buckler ES, et al. A robust, simple genotyping-by-sequencing (GBS) approach for high diversity species. PLoS One. 2011; 6(5):e19379. doi: 10.1371/journal.pone.0019379.

30. Glaubitz JC, Casstevens TM, Lu F, Harriman J, Elshire RJ, Sun Q, et al. TASSEL-GBS: a high capacity genotyping by sequencing analysis pipeline. PLoS One. 2014; 9(2): e90346. doi:10.1371/journal.pone.0090346.

31. Sun F, Fan G, Hu Q, Zhou Y, Guan M, Tong C, et al. The high-quality genome of *Brassica napus* cultivar 'ZS 11'reveals the introgression history in semi-winter morphotype. Plant J. 2017; 92(3):452–68. doi:10.1111/tpj.13669.

32. Langmead B, Salzberg SL. Fast gapped-read alignment with Bowtie 2. Nat Methods. 2012; 9(4): 357-359. doi:10.1038/nmeth.1923.

33. Danecek P, Auton A, Abecasis G, Albers CA, Banks E, DePristo MA, et al. The variant call format and VCFtools. Bioinformatics. 2011; 27(15):2156–8. doi:10.1093/bioinformatics/btr330.

34. Bradbury PJ, Zhang Z, Kroon DE, Casstevens TM, Ramdoss Y, Buckler ES. TASSEL: software for association mapping of complex traits in diverse samples. Bioinformatics. 2007; 23(19):2633–5. doi:10.1093/bioinformatics/btm308.

35. Pritchard JK, Stephens M, Donnelly P. Inference of population structure using multilocus genotype data. Genetics. 2000; 155(2): 945–59.

36. Evanno G, Regnaut S, Goudet J. Detecting the number of clusters of individuals using the software STRUCTURE: a simulation study. Mol Ecol. 2005; 14(8):2611–20.

37. Earl DA, vonHoldt BM. STRUCTURE HARVESTER: A website and program for visualizing STRUCTURE output and implementing the Evanno method. Conserv Genet Resour. 2012; 4: 359–361. doi:10.1007/s12686-011-9548-7.

38. Jakobsson M, Rosenberg NA. CLUMPP: a cluster matching and permutation program for dealing with label switching and multimodality in analysis of population structure. Bioinformatics. 2007; 23(14):1801–6.

39. Ramasamy RK, Ramasamy S, Bindroo BB, Naik VG. STRUCTURE PLOT: a program for drawing elegant STRUCTURE bar plots in user friendly interface. Springerplus. 2014; 3(1):431. doi:10.1186/2193-1801-3-431.

40. Kumar S, Stecher G, Li M, Knyaz C, Tamura K. MEGA X: molecular evolutionary genetics analysis across computing platforms. Mol Biol Evol. 2018; 35(6):1547–9.

41. Rambaut A, FigTree V. 1.4. 4. [Internet]. 2018; [Available from: http://tree.bio.ed.ac.uk/software/figtree]

42. Excoffier L, Lischer HEL. Arlequin suite ver 3.5: a new series of programs to perform population genetics analyses under Linux and Windows. Mol Ecol Resour. 2010; 10(3): 564–7.

43. Peakall R, Smouse PE. GenAlEx 6.5: genetic analysis in Excel. Population genetic software for teaching and research—an update. Bioinformatics [Internet]. 2012 Jul 20; 28(19):2537–9. Available from: https://doi.org/10.1093/bioinformatics/bts460

44. Slate J, Marshall T, Pemberton J. A retrospective assessment of the accuracy of the paternity inference program CERVUS. Mol Ecol. 2000; 9(6):801–8.

45. Kim B, Beavis WD. Numericware i: Identical by State Matrix Calculator. Evol Bioinforma. 2017; 13:1176934316688663.

46. Gu Z, Eils R, Schlesner M. Complex heatmaps reveal patterns and correlations in multidimensional genomic data. Bioinformatics. 2016; 32(18): 2847–9.

47. R Core Team. R: A Language and Environment for Statistical Computing [Internet]. Vienna, Austria; 2019. Available from: https://www.r-project.org/

48. Zhang C, Dong SS, Xu JY, He WM, Yang TL. PopLDdecay: A fast and effective tool for linkage disequilibrium decay analysis based on variant call format files. Bioinformatics. 2019; 35: 1786-1788. doi:10.1093/bioinformatics/bty875.

49. Purcell S, Neale B, Todd-Brown K, Thomas L, Ferreira MAR, Bender D, et al. PLINK: A tool set for whole-genome association and population-based linkage analyses. Am J Hum Genet. 2007; 81: 559-575. doi:10.1086/519795.

50. Gabriel SB, Schaffner SF, Nguyen H, Moore JM, Roy J, Blumenstiel B, et al. The structure of haplotype blocks in the human genome. Science. 2002; 296: 2225-2229. doi:10.1126/science.1069424.

51. Ganal MW, Durstewitz G, Polley A, Bérard A, Buckler ES, Charcosset A, et al. A large maize (zea mays L.) SNP genotyping array: Development and germplasm genotyping, and genetic mapping to compare with the B73 reference genome. PLoS One. 2011; 6: e28334. doi:10.1371/journal.pone.0028334.

52. Riedelsheimer C, Lisec J, Czedik-Eysenberg A, Sulpice R, Flis A, Grieder C, et al. Genome-wide association mapping of leaf metabolic profiles for dissecting complex traits in maize. Proc Natl Acad Sci U S A. 2012; 109: 8872–8877. doi:10.1073/pnas.1120813109.

53. Mandel JR, Nambeesan S, Bowers JE, Marek LF, Ebert D, Rieseberg LH, et al. Association Mapping and the Genomic Consequences of Selection in Sunflower. PLoS Genet. 2013; 9: e1003378. doi:10.1371/journal.pgen.1003378.

54. Chen R, Shimono A, Aono M, Nakajima N, Ohsawa R, Yoshioka Y. Genetic diversity and population structure of feral rapeseed (*Brassica napus* L.) in Japan. PLoS One. 2020; 15: e0227990. doi:10.1371/journal.pone.0227990.

55. Wu J, Li F, Xu K, Gao G, Chen B, Yan G, et al. Assessing and broadening genetic diversity of a rapeseed germplasm collection. Breed Sci. 2014; 64(4): 321–30.

56. Ahmad R, Quiros CF, Rahman H, Swati ZA, others. Genetic diversity analyses of *Brassica napus* accessions using SRAP molecular markers. Plant Genet Resour. 2014; 12(1):14.

57. Rafalski A. Applications of single nucleotide polymorphisms in crop genetics. Current Opinion in Plant Biology. 2002; 5: 94–100. doi:10.1016/S1369-5266(02)00240-6.

58. Bus A, Hecht J, Huettel B, Reinhardt R, Stich B. High-throughput polymorphism detection and genotyping in *Brassica napus* using next-generation RAD sequencing. BMC Genomics. 2012; 13(1):1–11.

59. Clarke WE, Parkin IA, Gajardo HA, Gerhardt DJ, Higgins E, Sidebottom C, et al. Genomic DNA enrichment using sequence capture microarrays: a novel approach to discover sequence nucleotide polymorphisms (SNP) in *Brassica napus* L. PLoS One. 2013; 8(12):e81992.

60. Huang S, Deng L, Guan M, Li J, Lu K, Wang H, et al. Identification of genome-wide single nucleotide polymorphisms in allopolyploid crop *Brassica napus*. BMC Genomics. 2013; 14(1):717.

61. Mantello CC, Cardoso-Silva CB, da Silva CC, de Souza LM, Junior EJS, de Souza Gonçalves P, et al. De novo assembly and transcriptome analysis of the rubber tree (*Hevea brasiliensis*) and SNP markers development for rubber biosynthesis pathways. PLoS One. 2014; 9(7):e102665.

62. Yang H, Wei C-L, Liu H-W, Wu J-L, Li Z-G, Zhang L, et al. Genetic divergence between *Camellia sinensis* and its wild relatives revealed via genome-wide SNPs from RAD sequencing. PLoS One. 2016; 11(3):e0151424.

63. Luo Z, Brock J, Dyer JM, Kutchan T, Schachtman D, Augustin M, et al. Genetic diversity and population structure of a *Camelina sativa* spring panel. Front Plant Sci. 2019; 10:184. doi:10.3389/fpls.2019.00184.

64. Hoque A, Fiedler JD, Rahman M. Genetic diversity analysis of a flax (*Linum usitatissimum* L.) global collection. BMC Genomics. 2020; 21:557. doi:10.1186/s12864-020-06922-2.

65. Luo Z, Iaffaldano BJ, Zhuang X, Fresnedo-Ramirez J, Cornish K. Analysis of the first Taraxacum kok-saghyz transcriptome reveals potential rubber yield related SNPs. Sci Rep. 2017; 7(1): 9939.

66. Shete S, Tiwari H, Elston RC. On estimating the heterozygosity and polymorphism information content value. Theor Popul Biol. 2000; 57(3): 265–71.

67. Eltaher S, Sallam A, Belamkar V, Emara HA, Nower AA, Salem KFM, et al. Genetic diversity and population structure of F3: 6 nebraska winter wheat genotypes using genotyping-by-sequencing. Front Genet. 2018; 9:76. doi:10.3389/fgene.2018.00076.

68. Ajala SO, Olayiwola MO, Ilesanmi OJ, Gedil M, Job AO, Olaniyan AB. Assessment of genetic diversity among low-nitrogen-tolerant early generation maize inbred lines using SNP markers. South African J Plant Soil. 2019; 36(3):181–8. doi:10.1080/02571862.2018.1537010.

69. Singh N, Agarwal N, Yadav HK. Genome-wide SNP-based diversity analysis and association mapping in linseed (*Linum usitatissimum* L.). Euphytica. 2019; 215(8):139.

70. Razak SA, Azman NHEN, Kamaruzaman R, Saidon SA, Yusof MFM, Ismail SN, et al. Genetic diversity of released malaysian rice varieties based on single nucleotide polymorphism markers. Czech J Genet Plant Breed. 2020; 56: 62–70. doi:10.17221/58/2019-CJGPB.

71. Coates BS, Sumerford D V, Miller NJ, Kim KS, Sappington TW, Siegfried BD, et al. Comparative performance of single nucleotide polymorphism and microsatellite markers for population genetic analysis. J Hered. 2009; 100(5): 556–64.

72. Diers BW, Osborn TC. Genetic diversity of oilseed Brassica napus germ plasm based on restriction fragment length polymorphisms. Theor Appl Genet. 1994; 88: 662–668. doi:10.1007/BF01253968.

73. Hasan M, Seyis F, Badani AG, Pons-Kühnemann J, Friedt W, Lühs W, et al. Analysis of genetic diversity in the *Brassica napus* L. gene pool using SSR markers. Genet Resour Crop Evol. 2006; 53: 793–802. doi:10.1007/s10722-004-5541-2.

74. Gazave E, Tassone EE, Ilut DC, Wingerson M, Datema E, Witsenboer HMA, et al. Population genomic analysis reveals differential evolutionary histories and patterns of diversity across subgenomes and subpopulations of *Brassica napus* L. Front Plant Sci. 2016; 7:525. doi:10.3389/fpls.2016.00525.

75. Yuan M, Zhou Y, Liu D. Genetic diversity among populations and breeding lines from recurrent selection in *Brassica napus* as revealed by RAPD markers. Plant Breed. 2004; 123: 9-12. doi:10.1046/j.0179-9541.2003.00903.x.

76. Li L, Chokchai W, Huang X, Huang T, Huang T, Li Q, et al. Comparison of AFLP and SSR for Genetic Diversity Analysis of *Brassica napus* Hybrids. J Agric Sci. 2011; 3: 101-110. doi:10.5539/jas.v3n3p101.

77. Gyawali S, Hegedus DD, Parkin IAP, Poon J, Higgins E, Horner K, et al. Genetic diversity and population structure in a world collection of *Brassica napus* accessions with emphasis on South Korea, Japan, and Pakistan. Crop Sci. 2013; 53, 1537-1545. doi:10.2135/cropsci2012.10.0614.

78. Morinaga T. Preliminary Note on Interspecific Hybridization in Brassica. Proc Imp Acad. 1929; 4: 620–622. doi:10.2183/pjab1912.4.620.

79. Cheung F, Trick M, Drou N, Lim YP, Park JY, Kwon SJ, et al. Comparative analysis between homoeologous genome segments of *Brassica napus* and its progenitor species reveals extensive sequence-level divergence. Plant Cell. 2009; 21: 1912–1928. doi:10.1105/tpc.108.060376.

80. Gómez-Campo C, Prakash S. 2 Origin and domestication. Dev Plant Genet Breed. 1999; 4: 33–58. doi:10.1016/S0168-7972(99)80003-6.

81. Xiao Y, Chen L, Zou J, Tian E, Xia W, Meng J. Development of a population for substantial new type *Brassica napus* diversified at both A/C genomes. Theor Appl Genet. 2010; 121(6): 1141–50.

82. Monfared MA, Samsampour D, Sharifi-Sirchi GR, Sadeghi F. Assessment of genetic diversity in *Salvadora persica* L. based on inter simple sequence repeat (ISSR) genetic marker. J Genet Eng Biotechnol. 2018; 16(2):661–7.

83. Tajima F. Statistical method for testing the neutral mutation hypothesis by DNA polymorphism. Genetics. 1989; 123(3):585–95.

84. Suzuki Y. Statistical methods for detecting natural selection from genomic data. Genes and Genetic Systems. 2010; 85: 359-376. doi:10.1266/ggs.85.359.

85. Torati LS, Taggart JB, Varela ES, Araripe J, Wehner S, Migaud H. Genetic diversity and structure in Arapaima gigas populations from Amazon and Araguaia-Tocantins river basins. BMC Genet. 2019; 20(1):13. doi:10.1186/s12863-018-0711-y.

86. Wright S. The interpretation of population structure by F-statistics with special regard to systems of mating. Evolution (N Y). 1965; 19(3):395–420.

87. Xiao Y, Cai D, Yang W, Ye W, Younas M, Wu J, et al. Genetic structure and linkage disequilibrium pattern of a rapeseed (*Brassica napus* L.) association mapping panel

revealed by microsatellites. Theor Appl Genet. 2012; 125, 437-447. doi:10.1007/s00122-012-1843-5.

88. Liu S, Fan C, Li J, Cai G, Yang Q, Wu J, et al. A genome-wide association study reveals novel elite allelic variations in seed oil content of *Brassica napus*. Theor Appl Genet. 2016; 129: 1203–1215. doi:10.1007/s00122-016-2697-z.

89. Chen R, Hara T, Ohsawa R, Yoshioka Y. Analysis of genetic diversity of rapeseed genetic resources in Japan and core collection construction. Breed Sci. 2017; 67: 239-247. doi:10.1270/jsbbs.16192.

90. Qian W, Sass O, Meng J, Li M, Frauen M, Jung C. Heterotic patterns in rapeseed (*Brassica napus* L.): I. Crosses between spring and Chinese semi-winter lines. Theor Appl Genet. 2007; 115(1):27–34.

91. Bus A, Körber N, Snowdon RJ, Stich B. Patterns of molecular variation in a species-wide germplasm set of *Brassica napus*. Theor Appl Genet. 2011; 123: 1413–1423. doi:10.1007/s00122-011-1676-7.

92. Flad DWF. Use of Rutabaga (*Brassica napus* var. *napobrassica*) for the Improvement of Canadian Spring Canola (*Brassica napus*). [Master’s thesis]. [Alberta (CA)]: University of Alberta. 2016; doi: 10.7939/R3319S744.

93. Shiranifar B, Hobson N, Kebede B, Yang RC, Rahman H. Potential of rutabaga (*Brassica napus* var. *napobrassica*) gene pool for use in the breeding of *B. napus* canola. Crop Sci. 2020; 60: 157-171. doi:10.1002/csc2.20074.

94. Cruz VM V., Luhman R, Marek LF, Rife CL, Shoemaker RC, Brummer EC, et al. Characterization of flowering time and SSR marker analysis of spring and winter type

Brassica napus L. germplasm. Euphytica. 2007; 153: 43-57. doi:10.1007/s10681-006-9233-1.

95. Malmberg MM, Shi F, Spangenberg GC, Daetwyler HD, Cogan NOI. Diversity and genome analysis of australian and global oilseed *Brassica napus* L. Germplasm using transcriptomics and whole genome re-sequencing. Front Plant Sci. 2018; 9:508. doi:10.3389/fpls.2018.00508.

96. Jakobsson M, Edge MD, Rosenberg NA. The relationship between FST and the frequency of the most frequent allele. Genetics. 2013; 193: 515-528. doi:10.1534/genetics.112.144758.

97. Bernardo R, Romero-Severson J, Ziegle J, Hauser J, Joe L, Hookstra G, et al. Parental contribution and coefficient of coancestry among maize inbreds: pedigree, RFLP, and SSR data. Theor Appl Genet. 2000; 100(3–4): 552–6.

98. Goode EL. Linkage Disequilibrium. In: Schwab M, editor. Encyclopedia of Cancer [Internet]. Berlin, Heidelberg: Springer Berlin Heidelberg; 2011. p. 2043–8. Available from: https://doi.org/10.1007/978-3-642-16483-5_3368

99. Guryev V, Smits BMG, Van De Belt J, Verheul M, Hubner N, Cuppen E. Haplotype block structure is conserved across mammals. PLoS Genet. 2006; 2: e121. doi:10.1371/journal.pgen.0020121.

100. Reich DE, Cargili M, Boik S, Ireland J, Sabeti PC, Richter DJ, et al. Linkage disequilibrium in the human genome. Nature. 2001; 411: 199–204. doi:10.1038/35075590.

101. Kim S, Plagnol V, Hu TT, Toomajian C, Clark RM, Ossowski S, et al. Recombination and linkage disequilibrium in Arabidopsis thaliana. Nat Genet. 2007; 39: 1151–1155. doi:10.1038/ng2115.

102. Sachs MM. Cereal germplasm resources. Plant Physiology. 2009; 149: 148–151. doi:10.1104/pp.108.129205.

103. Brachi B, Morris GP, Borevitz JO. Genome-wide association studies in plants: The missing heritability is in the field. Genome Biology. 2011; 12: 232. doi:10.1186/gb-2011-12-10-232.

104. Jia G, Huang X, Zhi H, Zhao Y, Zhao Q, Li W, et al. A haplotype map of genomic variations and genome-wide association studies of agronomic traits in foxtail millet (*Setaria italica*). Nat Genet. 2013; 45: 957–961. doi:10.1038/ng.2673.

105. Rakow G, Woods DL. Outcrossing in rape and mustard under saskatchewan prairie conditions. Can J Plant Sci. 1987; 67: 147–151. doi:10.4141/cjps87-017.

106. Becker HC, Damgaard C, Karlsson B. Environmental variation for outcrossing rate in rapeseed (*Brassica napus*). Theor Appl Genet. 1992; 84: 303-306. doi:10.1007/BF00229487.

107. Cuthbert JL, McVetty PBE. Plot-to-plot, row-to-row and plant-to-plant outcrossing studies in oilseed rape. Can J Plant Sci. 2001; 81: 657–664. doi:10.4141/P01-070.

108. Wu Z, Wang B, Chen X, Wu J, King GJ, Xiao Y, et al. Evaluation of linkage disequilibrium pattern and association study on seed oil content in Brassica napus using ddRAD sequencing. PLoS One. 2016; 11: e0146383. doi:10.1371/journal.pone.0146383.

109. Zhou Q, Zhou C, Zheng W, Mason AS, Fan S, Wu C, et al. Genome-wide SNP markers based on SLAF-seq uncover breeding traces in rapeseed (*Brassica napus* L.). Front Plant Sci. 2017; 8: 648. doi:10.3389/fpls.2017.00648.

110. Gao H, Ye S, Wu J, Wang L, Wang R, Lei W, et al. Genome-wide association analysis of aluminum tolerance related traits in rapeseed (*Brassica napus* L.) during germination. Genet Resour Crop Evol. 2020; doi:10.1007/s10722-020-00989-2.

111. Collins FS, Green ED, Guttmacher AE, Guyer MS. A vision for the future of genomics research. Nature. 2003; 422: 835-847. doi:10.1038/nature01626.

Supporting information

S1 Table. List of the genotypes analyzed in this study.

S2 Table. Marker diversity parameters.

S3 Table. Subpopulation-wise marker diversity parameters.

S4 Table. (a) Percentage of variation explained by the first 3 axes, (b) Eigen values by axis and sample eigen vectors.

S5 Table. Kinship matrix.

S6 Table. Mean LD values according to distance.

S7 Table. Subpopulation-wise and chromosome-wise LD decay rate (Kb) within each subpopulation.

S8 Table. Subpopulation specific and common haplotype blocks.

S1 Fig. Histogram of IBS coefficients.

S2 Fig. Chromosome-wise LD decay rate (Kb) in A genome considering whole collection.

S3 Fig. Chromosome-wise LD decay rate (Kb) in C genome considering whole collection.

Click here to access/download;Figure;Fig 1.tif

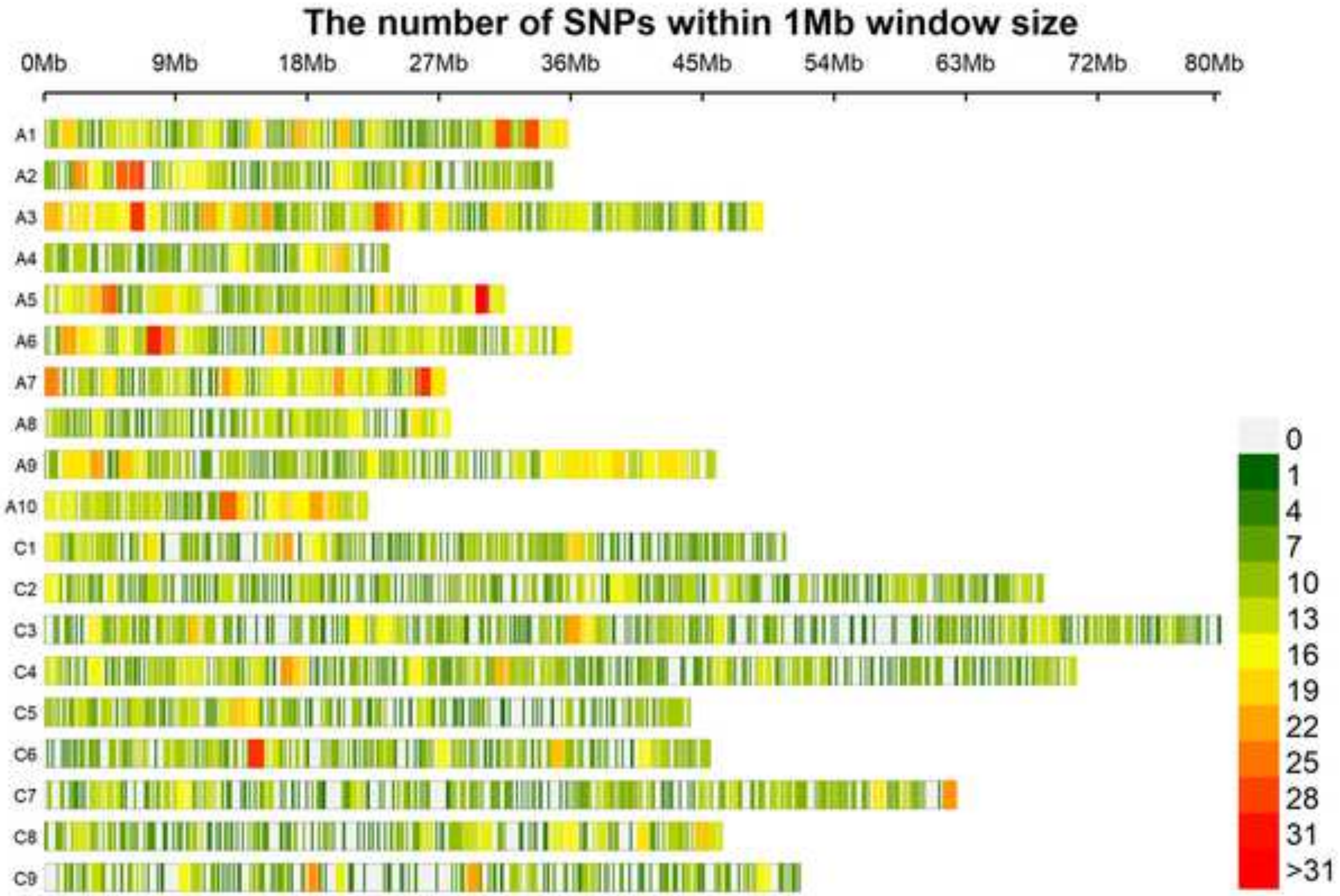

Click here to access/download;Figure;Fig 2.tif

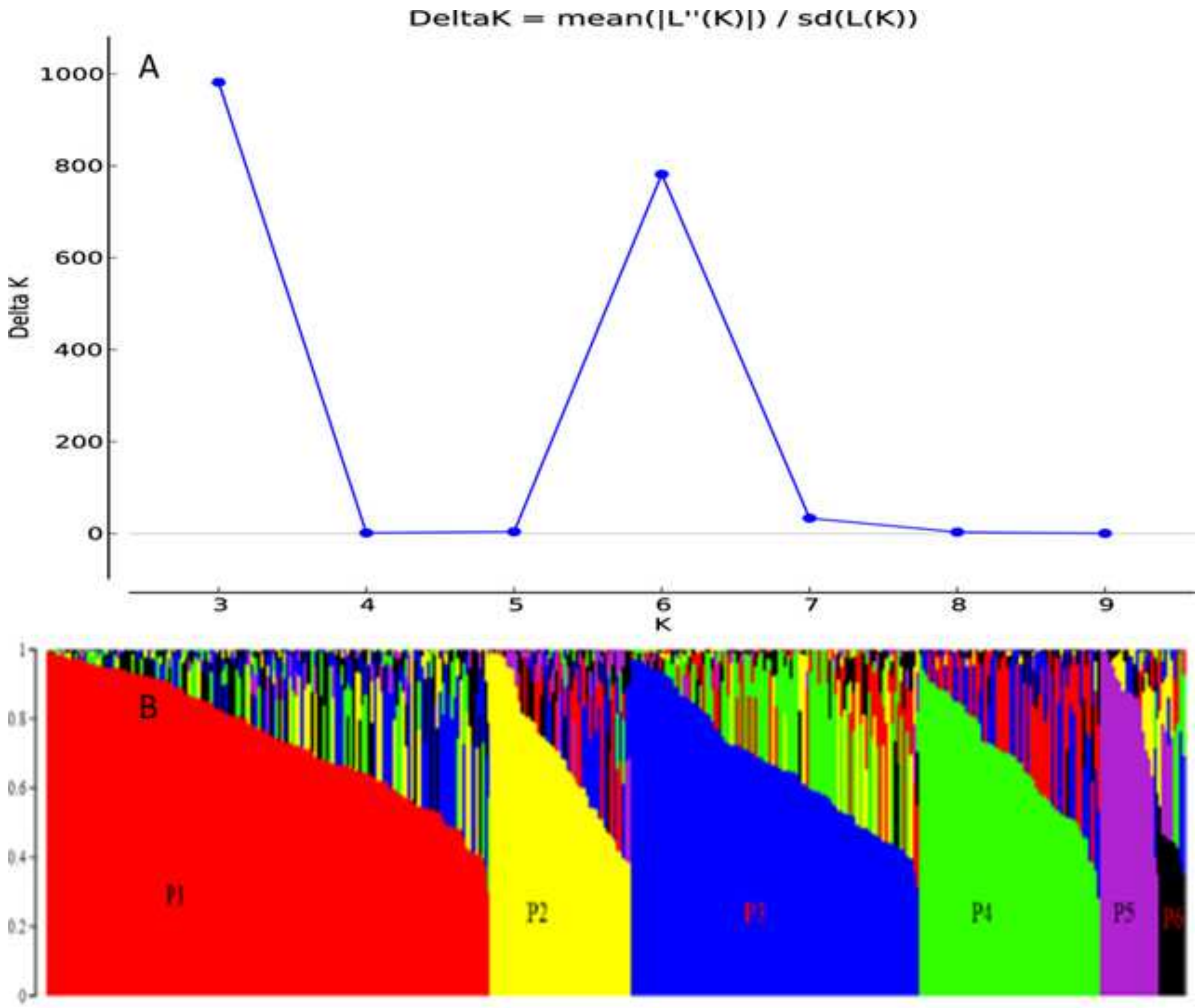

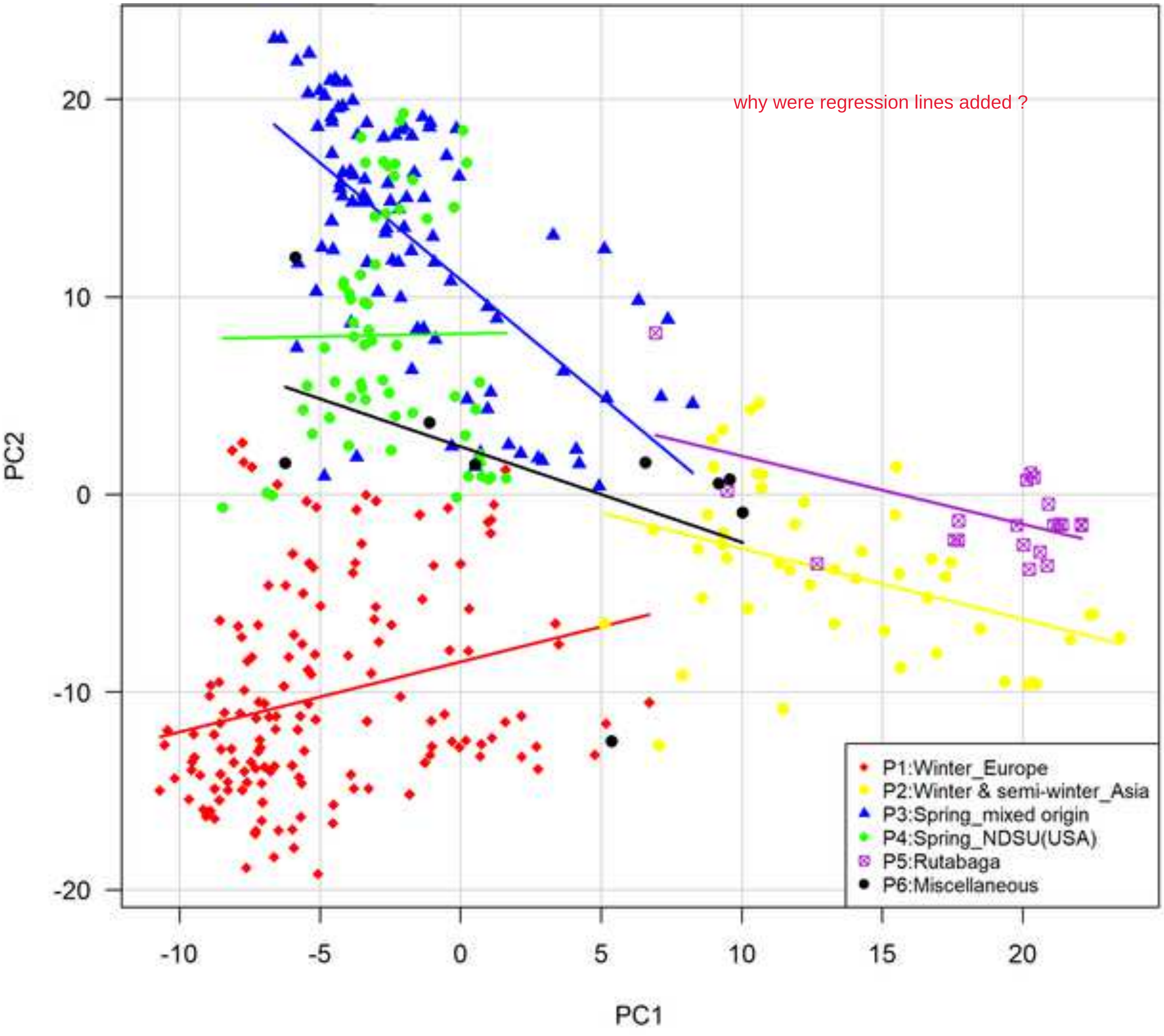
why were regression lines added ?
PC2
PC1
20
10
0
-10
-20
-10
-5
0
5
10
15
20
P1:Winter_Europe
P2:Winter & semi-winter_Asia
P3:Spring_mixed origin
P4:Spring_NDSU(USA)
P5:Rutabaga
P6:Miscellaneous

Click here to access/download;Figure;Fig 4.tif

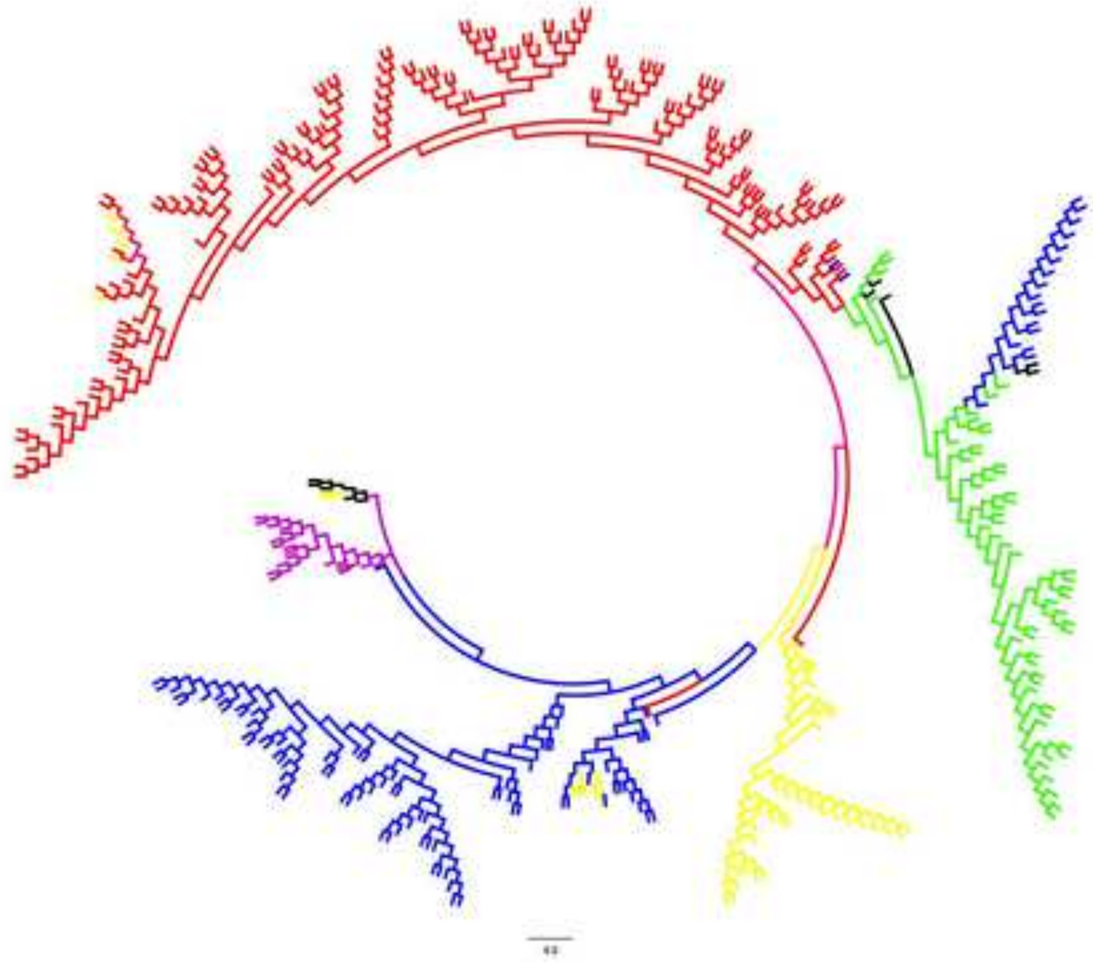

Click here to access/download;Figure;Fig 5.tif

can you add on the picture information referring to P1, P2, P3; P4 and P5 groups ?

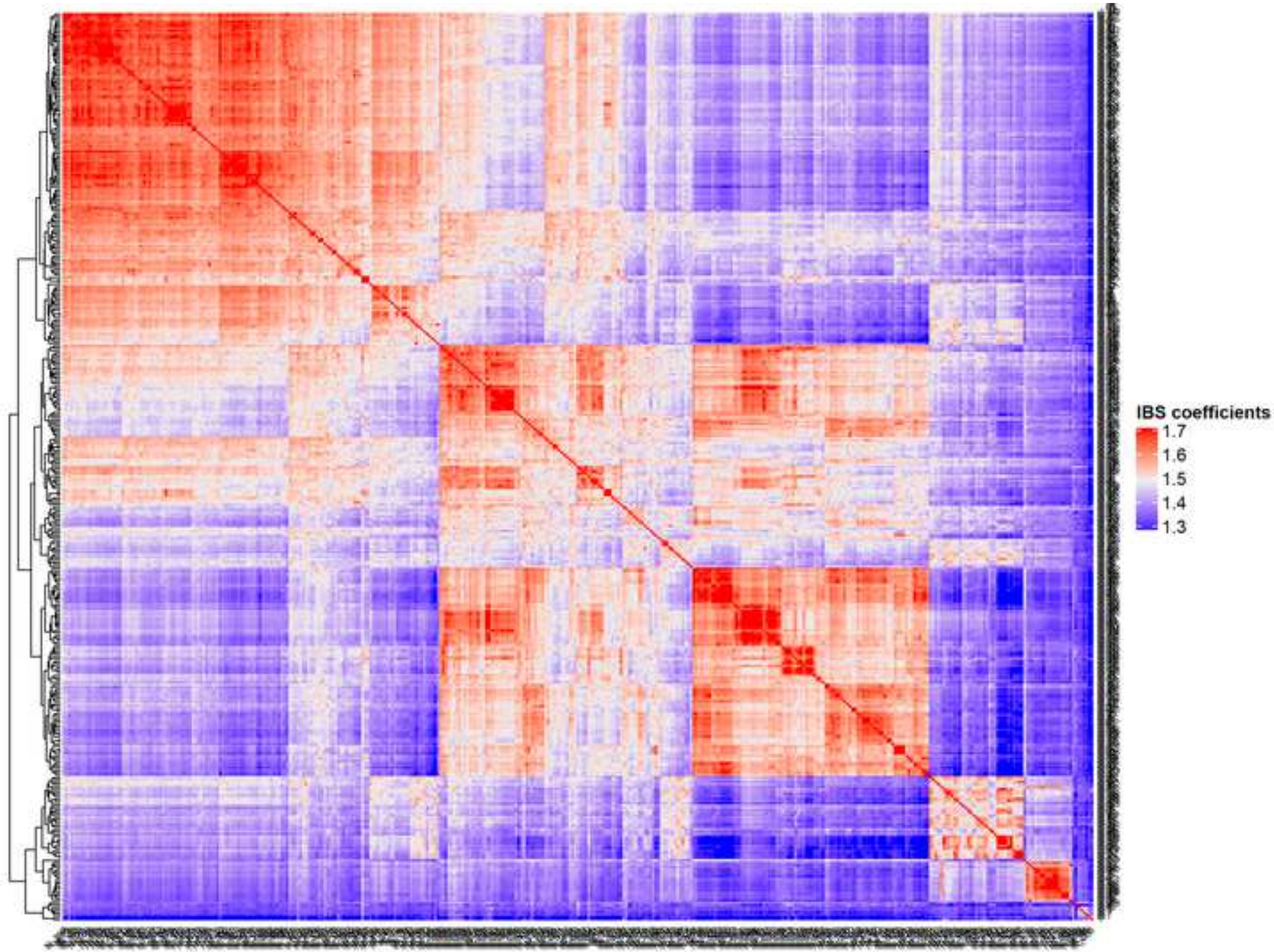

Click here to access/download;Figure;Fig 6.tif

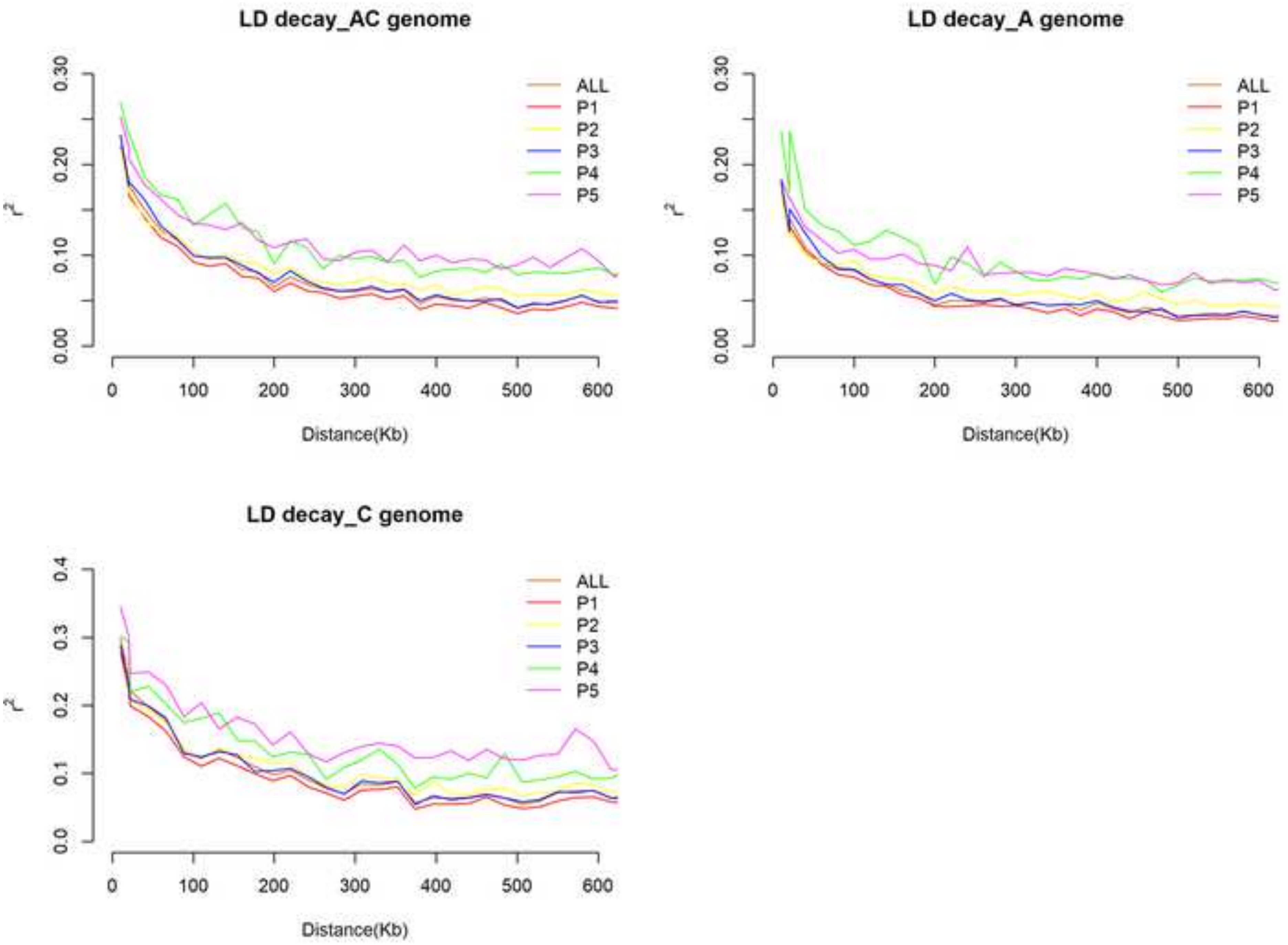

Click here to access/download
Supporting Information
Declarations_Funding_Author contribution_Data availability.docx

Click here to access/download
Supporting Information
S1 Table.xlsx

Click here to access/download
Supporting Information
S1_Fig.tiff

Click here to access/download
Supporting Information
S2 Table.xlsx

Click here to access/download
Supporting Information
S2_Fig.tiff

Click here to access/download
Supporting Information
S3 Table.xlsx

Click here to access/download
Supporting Information
S3_Fig.tiff

Click here to access/download
Supporting Information
S4 Table.xlsx

Click here to access/download
Supporting Information
S5 Table.xlsx

Click here to access/download
Supporting Information
S6 Table.xlsx

Click here to access/download
Supporting Information
S7 Table.xlsx

Click here to access/download
Supporting Information
S8 Table.xlsx
