# Supplementary material for: Linkage disequilibrium and population structure in a core collection of Brassica napus (L.)
Source: PLoS One. 2022 Mar 1;17(3):e0250310. doi: 10.1371/journal.pone.0250310 (PMC8887726; doi:10.1371/journal.pone.0250310)
Supplement: Attachment — Submitted filename: Response to editor and reviewer.docx [file pone.0250310.s012.docx]

Journal Requirements:

When submitting your revision, we need you to address these additional requirements.

1. Please ensure that your manuscript meets PLOS ONE's style requirements, including those for file naming.

**Response**: We have checked and tried our best to format the manuscript according to the PLOS ONE’s style.

2. We note that you have indicated that data from this study are available upon request. PLOS only allows data to be available upon request if there are legal or ethical restrictions on sharing data publicly. For more information on unacceptable data access restrictions, please see http://journals.plos.org/plosone/s/data-availability#loc-unacceptable-data-access-restrictions.

 In your revised cover letter, please address the following prompts:

2a) If there are ethical or legal restrictions on sharing a de-identified data set, please explain them in detail (e.g., data contain potentially sensitive information, data are owned by a third-party organization, etc.) and who has imposed them (e.g., an ethics committee). Please also provide contact information for a data access committee, ethics committee, or other institutional body to which data requests may be sent.

**Response**: No restriction

2b) If there are no restrictions, please upload the minimal anonymized data set necessary to replicate your study findings as either Supporting Information files or to a stable, public repo-sitory and provide us with the relevant URLs, DOIs, or accession numbers. For a list of acceptable repositories, please see <http://journals.plos.org/plosone/s/data-availability#loc-recommended-repositories>.

**Response**: Datasets are available online.

3. Thank you for stating the following financial disclosure:

The funders had no role in study design, data collection and analysis, decision to publish, or preparation of the manuscript.

At this time, please address the following queries:

a) Please clarify the sources of funding (financial or material support) for your study. List the grants or organizations that supported your study, including funding received from your institution.

**Response**: The study was funded by the U.S. Department of Agriculture - National Institute of Food and Agriculture (Hatch Project No. ND01581).

b) State what role the funders took in the study. If the funders had no role in your study, please state: “The funders had no role in study design, data collection and analysis, decision to publish, or preparation of the manuscript.”

**Response**: The funders had no role in study design, data collection and analysis, decision to publish, or preparation of the manuscript.

c) If any authors received a salary from any of your funders, please state which authors and which funders.

**Response**: No salary received.

d) If you did not receive any funding for this study, please state: “The authors received no specific funding for this work.”

**Response**: Not applicable.

4. Thank you for submitting the above manuscript to PLOS ONE. During our internal evaluation of the manuscript, we found significant text overlap between your submission and the following previously published works, some of which you are an author.

https://bmcgenomics.biomedcentral.com/articles/10.1186/s12864-020-06922-2

We would like to make you aware that copying extracts from previous publications, especially outside the methods section, word-for-word is unacceptable. In addition, the reproduction of text from published reports has implications for the copyright that may apply to the publications.

Please revise the manuscript to rephrase the duplicated text, cite your sources, and provide details as to how the current manuscript advances on previous work. Please note that further consideration is dependent on the submission of a manuscript that addresses these concerns about the overlap in text with published work.

**Response**: We have rephrased the duplicated text in revised throughout the manuscript.

**Reviewers' comments:**

Reviewer's Responses to Questions

**Comments to the Author**

1. Is the manuscript technically sound, and do the data support the conclusions?

The manuscript must describe a technically sound piece of scientific research with data that supports the conclusions. Experiments must have been conducted rigorously, with appropriate controls, replication, and sample sizes. The conclusions must be drawn appropriately based on the data presented.

Reviewer #1: No

Reviewer #2: Partly

Reviewer #3: Yes

**Response:** We edited revised manuscript to solve these issues.

2. Has the statistical analysis been performed appropriately and rigorously?

Reviewer #1: No

Reviewer #2: Yes

Reviewer #3: Yes

3. Have the authors made all data underlying the findings in their manuscript fully available?

The [PLOS Data policy](http://www.plosone.org/static/policies.action#sharing) requires authors to make all data underlying the findings described in their manuscript fully available without restriction, with rare exception (please refer to the Data Availability Statement in the manuscript PDF file). The data should be provided as part of the manuscript or its supporting information, or deposited to a public repository. For example, in addition to summary statistics, the data points behind means, medians and variance measures should be available. If there are restrictions on publicly sharing data—e.g. participant privacy or use of data from a third party—those must be specified.

Reviewer #1: Yes

Reviewer #2: No

Reviewer #3: No

**Response**: Data are available online

4. Is the manuscript presented in an intelligible fashion and written in standard English?

PLOS ONE does not copyedit accepted manuscripts, so the language in submitted articles must be clear, correct, and unambiguous. Any typographical or grammatical errors should be corrected at revision, so please note any specific errors here.

Reviewer #1: No

Reviewer #2: No

Reviewer #3: Yes

Response: We edited and rearranged text in revised manuscript to solve these issues.

5. Review Comments to the Author

**Reviewer #1**: The authors proposed to analyse the genetic diversity present in a collection of 383 accessions of Brassica napus representing genotypes present worldwilde and aims at characterizing the genepool present in US breeding programs. This seems quite interesting and the size of of the populations should be sufficient to draw interesting conclusions. However hypotheses and research questions are not clearly expressed at the end of the introduction, and too many extra data are provided and not used in the manuscript (for instance flowering time data). Moreover, many analyses are performed , but may be too much without really refering to a global strategy and the global aim of each batch of analyses.

Detailed analysis and questions are reported in the attached file
A rapid check at table S1 showed some problems considering geogagraphic origins of different accessions
for instance Lembkes, Krapphauser, fertodi => Germany instead of South Korea
Dramor (Poland) should be Darmor (France) I think
Bienvenu (USA) should be Bienvenu (France)
Jet-neuf (Canada) should be Jet-neuf (France)
.... I didn't check carefully all accessions but these mistakes may lead to inappropriate interpertations of subpopulations composition

**Response**: Thank you for pointing these out. In revised manuscript, we have tried to incorporate the suggestions of the reviewer to make the hypotheses and research questions more clear (at later part of introduction). We remove the flowering time data and clarified how the core collection was obtained (Line: 107-116 in manuscript). We think performed analyses strengthen each other as well as the objective.

In case of table S1, data was provided according to USDA (GRIN-Global) website (source: <https://npgsweb.ars-grin.gov/gringlobal/search>). We did a google search to know the origin of many cultivars and failed to get the information. USDA-GRIN recorded many genotypes on the basis of country of collection – and we used this recorded information in this manuscript. Therefore, to clear it out, we have written as “Country or origin/collected”.

Reviewer #2: The manuscript describes an attempt to study genetic diversity and population differentiation inherent in a germplasm assemblage of Brassica napus. Study is of interest for plant breeders engaged in the improvement of this important oilseed crop. Abstract and introduction are very poorly written. The method and material section must include some information about the procedure followed to maintain germplasm lines used for genotyping. In the absence of any information, one would suspect these to be the products of open pollination. Inherent heterozygosity can be a problem for genetic studies in any often cross-pollinated crop like B.napus. Filtering out of a large proportion of heterozygous SNPs may be the cause for the retention of only 8,502 SNPs out 497336 discovered initially. Structure analysis shows lot of admixing. Heterozygosity inherent in the germ plasm will affect all genetic inferences e.g. polymorphic loci, population variation, LD, genetic differentiation and even genetic diversity. It is always better to use homozygous lines. One may not like to use the term core collection for any germplasm assemblage. Authors may like to provide reference or provide details regarding method used to extract the core ( if it is actually a core collection) from a large germplasm base. In spite of these limitations, it was interesting to find clear geographic and growth habit related clustering from STRUCTURE analysis. The manuscript also suffers from narrative coherence.

**Response**: Thanks for your critical inputs. In revised manuscript, we have made the changes according to reviewer’s suggestions. We rewrite the introduction section to make it clear. To improve the narrative coherence, we edited almost all section in revised manuscript.

How core collection has been obtained and maintained, was described in materials and methods section (Line 107 to 116).

SNP number reduced, because of following strict filtering criteria (Line 134 to 139)

Reviewer #3: Manuscript PONE-D-21-09997 describes genetic diversity, population structure and LD in Brassica napus - the second major oilseed crop grown worldwide. Here are some comments:
This study does not provide details on sampling (how many plants were sampled for DNA isolation, how SNPs were scored codominant/dominant; how heterozygosity was handled etc). I could not see genotypic data of 383 accessions. Have authors submitted genotypic data to any public database repository or provided in a supplementary Table?

**Response**: Thank you for pointing these points. In revised manuscript, we have incorporated the information according to reviewer’s suggestion.

For DNA extraction, we used three leaf samples per genotype (Line 119)

For diversity analysis we do not need to score the codominant/dominant markers. To run structure, we scored the homozygous major, homozygous minor and heterozygous SNP as 1, 0 and 0.5 respectively using Tassel.

Data availability: GBS and SNP data are available at: PRJNA687906 (<https://www.ncbi.nlm.nih.gov/biosample/17159566>) and PRJEB42419 (https://[www.ebi.ac.uk/eva/?eva-study=PRJEB42419](http://www.ebi.ac.uk/eva/?eva-study=PRJEB42419)), respectively.

Reviewer #3: L172-173: Table 1 and Fig 1 does not support this claim.

**Response**: While we appreciate the reviewer’s feedback, we respectfully disagree. The SNP density was highest on chromosome A7 as every 71.1 kb distance contains at least one SNP and was lowest on chromosome C9 as every 134.5 kb distance contains at least one SNP.

Reviewer #3: L191-192: It is worthwhile to provide statistics of transitions and transversions across different chromosomes

**Response**: As our primary objective is not to describe the SNPs in details, we think statistics of transitions and transversions across genome is enough to give an idea to the reader.

Reviewer #3: L340: GBS is one of the approaches (Illumina SNP, resequencing, sequencing and DArTseq)

**Response**: Yes.

Reviewer #3: L360: There are no wild accessions of B. napus.

**Response**: Yes, we agree the reviewer’s comment. We deleted this information from revised manuscript

Reviewer #3: L460: Favorable alleles?

**Response**: We changed positive alleles to favorable alleles.

Reviewer #3: Fig 3: How much genetic variation is explained by PC1 and PC2? What these lines indicate?

**Response**: PC1 and PC2 explained 13.50% and 7.22% of variation respectively. Lines indicate regression. (Line 226)

Reviewer #3: Fig4: I could not see bootstrap values here. Please label all accessions (name or serial number 1 to 383 and support with supplementary Table)

**Response**: bootstrap value added. But did not add label as it make the figure congested.

Reviewer #3: Figure 5 is not legible. You may move to supplementary information.

**Response**: We would like to keep it as it is for the better understanding of readers.

Minor comments
Reviewer #3: L52: Use either rapeseed or canola (it is already described in L49)

Response: In revised manuscript, we used rapeseed.

Reviewer #3: L72: There are several studies to support collinearity between Arabidopsis and Brassica (see publications of Parkin and Chris Pires groups, https://doi.org/10.1111/pce.12644 etc)

**Response**: Yes.

Reviewer #3: L527: There are several reports. You meant NSDU accessions?

Response: Yes, we mean NDSU accessions i.e. NDSU advanced breeding lines.
