# Supplementary material for: Linkage disequilibrium and population structure in a core collection of Brassica napus (L.)
Source: PLoS One. 2022 Mar 1;17(3):e0250310. doi: 10.1371/journal.pone.0250310 (PMC8887726; doi:10.1371/journal.pone.0250310)
Supplement: Attachment — Submitted filename: Response to reviewer.docx [file pone.0250310.s014.docx]

# Summary: Reject and reconsider for publication after major revisions

The manuscript describes studies the population structure and genetic diversity of 383 *Brassica napus* (canola) accessions using 8502 SNP. The authors inferred six groups based on STRUCTURE, principal component analysis and NJ analyses. The manuscript is well written. However, the STRUCTURE analysis was not comprehensive enough. In addition, the interpretation of the results was not consistent with the Figures 2 and 3. Therefore, I recommend rejection of the MS and re-consideration for publication after major revisions.

# Major issues

1. **Introduction**

The literature cited by the authors are dated (2012 to 2014). More recent population studies on *B. napus* and other *Brassica* exist e.g., An et al. (2019) Nature Communications; Lu et al. (2019) Nature Communications and Yu et al (2021) BMC Genomics. The authors should include how their study will add to the current knowledge on genetic diversity of canola.

**Response**: We have added the references mentioned by the reviewer in revised manuscript. The study was done to exploit the diversity of NDSU canola core collection. The results will be used to enrich NDSU canola parental stock.

# 2. Materials and Methods

The authors run the STRUCTURE program using the admixture model at 10,000 burning period and 50,000 Monte Carlo Markov Chain. Yu et al (2021) showed that Puechmaille (2016) method (MedMedK, MedMeaK, MaxMedK and MaxMeaK statistics) was a better estimator of the number of clusters compared to the Evanno et al. (2005) method (DeltaK statistics) which was used by the authors. The authors should re-run STRUCTURE under different parameters to verify that the inference on population sub-populations is robust.

**Response**: We re-ran the structure analysis according to reviewer suggestion. We discussed it in methodology section (Line151 to line160).

# 3. Results

(a) A requirement for the STRUCTURE program is that markers be unlinked. Inclusion of multiple closely linked markers may have a large effect on calculation of population structure. Also, it is not known whether these SNPs are in non-coding region or are synonymous, to eliminate the bias introduced by selection when inferring population structure. The authors should comment on the appropriateness of using the selected SNP markers for their study.

**Response**: To break the linkage between markers we considered the markers which are located > 1000bp from each other (L145 to L146). We didn’t consider whether SNPs are synonymous or non- synonymous as dN/dS ratio is insensitive to selection pressure in intraspecific individuals or individual from same population (Kryazhimskiy and Plotkin, 2008).

(b) The authors did not interpret their STRUCTURE and PCA data correctly:
(i) Figure 2 shows that the maximum peak is at K=3. The second and smaller peak is at K=6. Therefore, the number of inferred clusters using STRUCTURE was likely 3 and not 6.
(ii) Figure 3-The Principal component analysis shows 3 clusters and not 6. Group 1-red (winter Europe),

Group 2 -green and blue (spring_mixed origin and spring_NDSU) and Group 3-violet and yellow (Rutabaga, winter and semi winter Asia)

NB: the miscellaneous group (black) which are scattered is in all 3 groups cannot be considered as a different subgroup (Authors own admission Lines 220-221).

**Response**: In revised manuscript, we corrected and discussed the figures according to reviewer suggestion (L218 to L237).

(c) The authors should indicate the NJ using rooted and unrooted trees. The authors should also indicate UPGMA rooted and unrooted trees.

**Response**: Unrooted NJ tree was used. We belief that Structure, PCA and NJ tree already addressed our targets.

(d) Please could the numbers on the trees be related back to the accession identifiers?

**Response**: Yes, We hope the provided new picture will clarify the issue.

(e) Some accessions had the same name or variation of it but different accession number. Some accessions were also advanced breeding lines. Can the authors comment on the genetic similarity of such accessions in the results section? Will these influence the calculation of population structure?

**Response**: Before formulating the core collection, we discarded the accession, which has same name with different PI number or same PI number with different name. So that we did not have any accession like that. The similarity among advanced breeding lines as well as among other genotypes has been discussed in discussion section.

# Discussion

The authors need to be update after the suggested changes.

**Response**: We updated the discussion section.

# Minor issues

1. Line 112-Did the authors use leaves from single plants or bulk plants?

**Response**: Bulk plants

2. Line 136-reword the core collection was divided into subgroups-Do you mean the number of sub-groups were inferred using STRUCTURE.

**Response**: Rewording done in revised manuscript.

**Reference**

Kryazhimskiy, S., and J.B. Plotkin. 2008. The population genetics of dN/dS. PLoS Genet. 4(12): e1000304.
