# Supplementary material for: Linkage disequilibrium and population structure in a core collection of Brassica napus (L.)
Source: PLoS One. 2022 Mar 1;17(3):e0250310. doi: 10.1371/journal.pone.0250310 (PMC8887726; doi:10.1371/journal.pone.0250310)
Supplement: Attachment — Submitted filename: Response to reviewer.docx [file pone.0250310.s015.docx]

6. Review Comments to the Author

Please use the space provided to explain your answers to the questions above. You may also include additional comments for the author, including concerns about dual publication, research ethics, or publication ethics. (Please upload your review as an attachment if it exceeds 20,000 characters)

Reviewer #2: The authors have revised the manuscript as per the suggestions by the reviewers. The revised manuscript can be accepted for publication.

**Response:** Thank you very much for accepting the manuscript.

Reviewer #5: Review of Rahman et al.

Rahman et al. analyzed the genotypes of 383 germplasms of Brassica napus, a part of which consists of NDSU core collection using the genotype by sequencing method. They identified population structures and evaluated the level of linkage disequilibrium. They also observed that the C genome have much higher level of LD than the A genome.

This manuscript has been revised twice, but this is the first time for me to review the paper. I would agree that the manuscript would provide valuable information for this research area and the data presented here are mostly well analyzed, but the manuscript lacks coordination in writing and still has a lot of typos, some of which would be described later. I therefore cannot recommend the manuscript to be published in PLOS ONE in its current form.

Major comments

1. Although the introduction part is very detailed. Some important information seems missing. In particular, it is not clear how the authors defined the five subpopulations is not clear to me. There is no description for rutabaga type, which would be difficult to understand for non-specialist of B. napus.

**Response**: We added description of rutabaga type in introduction (line 68 to 74). We assumed the subpopulation of collection according to their type and origin. Details has been added in materials and method section (line 129 to 134).

2. I initially thought that 8502 high-quality SNPs out of 497,336 unfiltered SNPs are too small, but probably the number is the one after the thinning. Please describe the number of high-quality SNPs before the thinning.

**Response**: We added the number (line 151 to 164) in materials and method section

3. There are several comparisons of statistics, but there are little statistical tests. For example, in the line 193, the authors stated that ts/tv ratio in A genome was higher than that in C genome, but the difference was not properly tested. So many of the analyses were more or less too descriptive.

**Response**: ts/tv ratio is a simple descriptive statistics and ratio of two numbers. It does not need any statistical test. Other statistical test such as Tajima's D (line 277: 1000 permutations), AMOVA (line 291: 1023 permutations, *p* ˂ 0.001), *Fst* comparisons (line 296: 1000 permutations, *p* ˂ 0.01), NJ (line 251: 1000 bootstraps) has been performed accordingly. Structure analysis has been done in details.

4. In the STRUCTURE analysis, I do not get what is the meaning of “accession assigned/unassigned”. STRUCTURE usually does not require any a priori assumption, in my understanding. Please add more description in the method section.

**Response**: Yes, structure does not require any a priori assumption. But as we divided the collection into subpopulation according to their type and origin, that’s why we ran structure considering accession assigned to specific subpopulation and accession unassigned to specific subpopulation to determine the exact cluster number of the collection. Details added in material and method section (line 166 to 182).

5. In the line 313, the meanings of “the mean linked LD”, “mean unlinked LD”, and “loci pair under linked LD”, are not clear to me. In the method section, the mean lined LD was calculated using SNPs with r2<0.2 (line 179), but it means they are unlinked. In addition, % is not added to 0.44, 0.02? It is somewhat confusing.

**Response**: That was typo. We changed it accordingly (line 197 to 205 and line 335 to 354).

6. In the line 401, the authors mentioned that the higher transition to transversion ratio is due to natural selection, but I do not agree with it. The pattern is ubiquitously found in many organisms including humans, and in non-coding regions, and people usually think that this is due to higher mutation rate of transition than transversion.

**Response**: We agreed to reviewer comment and deleted the information (line 431-432).

7. The sentence in the line 427-428 seems contradicting to the previous sentence. Probably the words, “homogeneity of the diversity indices” is not appropriate.

**Response**: We agreed to reviewer comment and deleted the information (line 457 to 459).

8. In the line 471, NJ is certainly distance-based method, but PCA is not.

**Response**: We agreed to reviewer comment and changed accordingly (line 503 to 504).

9. In the line 524, the authors mentioned that the level of LD in B. napus is low compared with the other crops, but there is no quantitative statement. I also wonder whether authors used the thinned data to calculate the mean LD. If ones remove the linked SNPs from the analysis, the level of LD obviously decreases.

**Response**: We modified the information according to reviewer comments (line 558 to 561 and line 574 to 577)

Minor comments,

1. L143: row SNPs would be raw SNPs
2. L153: burn-in length
3. L405: Rephrase the words “moderate or low informative”.
4. L446: the word “type” is not necessary?

**Response**: We tried our best to correct typos and changed accordingly (line 151, line 161, line 411 and line 450)

5. The assignment of Ks in Figure 4 is not clear. Are they determined using the presented tree?

**Response**: Each branch is color-coded according to genotype belongs to subpopulation P1 to P5. Genotypes were grouped into three clusters by dividing the tree using black solid lines according to structure output (line 250 to 253).
