# Supplementary material for: Linkage disequilibrium and population structure in a core collection of Brassica napus (L.)
Source: PLoS One. 2022 Mar 1;17(3):e0250310. doi: 10.1371/journal.pone.0250310 (PMC8887726; doi:10.1371/journal.pone.0250310)
Supplement: Attachment — Submitted filename: Reviewer Comments.pdf [file pone.0250310.s013.pdf]

Summary: Reject and reconsider for publication after major revisions

The manuscript describes studies the population structure and genetic diversity of 383 *Brassica napus* (canola) accessions using 8502 SNP. The authors inferred six groups based on STRUCTURE, principal component analysis and NJ analyses. The manuscript is well written. However, the STRUCTURE analysis was not comprehensive enough. In addition, the interpretation of the results was not consistent with the Figures 2 and 3. Therefore, I recommend rejection of the MS and re-consideration for publication after major revisions.

Major issues

1. Introduction

The literature cited by the authors are dated (2012 to 2014). More recent population studies on *B. napus* and other *Brassica* exist e.g., An et al. (2019) Nature Communications; Lu et al. (2019) Nature Communications and Yu et al (2021) BMC Genomics. The authors should include how their study will add to the current knowledge on genetic diversity of canola.

2. Materials and Methods

The authors run the STRUCTURE program using the admixture model at 10,000 burning period and 50,000 Monte Carlo Markov Chain. Yu et al (2021) showed that Puechmaille (2016) method (MedMedK, MedMeaK, MaxMedK and MaxMeaK statistics) was a better estimator of the number of clusters compared to the Evanno et al. (2005) method (DeltaK statistics) which was used by the authors. The authors should re-run STRUCTURE under different parameters to verify that the inference on population sub-populations is robust.

3. Results

(a) A requirement for the STRUCTURE program is that markers be unlinked. Inclusion of multiple closely linked markers may have a large effect on calculation of population structure. Also, it is not known whether these SNPs are in non-coding region or are synonymous, to eliminate the bias introduced by selection when inferring population structure. The authors should comment on the appropriateness of using the selected SNP markers for their study.

(b) The authors did not interpret their STRUCTURE and PCA data correctly:

(i) Figure 2 shows that the maximum peak is at K=3. The second and smaller peak is at K=6. Therefore, the number of inferred clusters using STRUCTURE was likely 3 and not 6.

(ii) Figure 3-The Principal component analysis shows 3 clusters and not 6.

Group 1-red (winter Europe),

Group 2 -green and blue (spring_mixed origin and spring_NDSU) and

Group 3-violet and yellow (Rutabaga, winter and semi winter Asia)

NB: the miscellaneous group (black) which are scattered is in all 3 groups cannot be considered as a different subgroup (Authors own admission Lines 220-221).

(c) The authors should indicate the NJ using rooted and unrooted trees.
The authors should also indicate UPGMA rooted and unrooted trees.

(d)Please could the numbers on the trees be related back to the accession identifiers?

(e) Some accessions had the same name or variation of it but different accession number. Some accessions were also advanced breeding lines. Can the authors comment on the genetic similarity of such accessions in the results section? Will these influence the calculation of population structure?

Discussion

The authors need to be update after the suggested changes.

Minor issues

1. Line 112-Did the authors use leaves from single plants or bulk plants?
2. Line 136-reword the core collection was divided into subgroups-Do you mean the number of sub-groups were inferred using STRUCTURE.
